# TET1 regulates gene expression and repression of endogenous retroviruses independent of DNA demethylation

**DOI:** 10.1093/nar/gkac642

**Published:** 2022-07-29

**Authors:** Paul Stolz, Angelo Salazar Mantero, Andrey Tvardovskiy, Enes Ugur, Lucas E Wange, Christopher B Mulholland, Yuying Cheng, Michael Wierer, Wolfgang Enard, Robert Schneider, Till Bartke, Heinrich Leonhardt, Simon J Elsässer, Sebastian Bultmann

**Affiliations:** Faculty of Biology and Center for Molecular Biosystems (BioSysM), Human Biology and BioImaging, Ludwig-Maximilians-Universität München, Munich 81377, Germany; Science for Life Laboratory, Department of Medical Biochemistry and Biophysics, Karolinska Institutet 17165 Stockholm, Sweden, Ming Wai Lau Centre for Reparative Medicine, Stockholm Node, Karolinska Institutet 17177 Stockholm, Sweden; Institute of Functional Epigenetics (IFE), Helmholtz Zentrum München, 85764 Neuherberg, Germany; Faculty of Biology and Center for Molecular Biosystems (BioSysM), Human Biology and BioImaging, Ludwig-Maximilians-Universität München, Munich 81377, Germany; Department of Proteomics and Signal Transduction, Max-Planck Institute of Biochemistry, Martinsried 82152, Germany; Faculty of Biology, Anthropology and Human Genomics, Ludwig-Maximilians-Universität München 82152, Planegg-Martinsried, Germany; Faculty of Biology and Center for Molecular Biosystems (BioSysM), Human Biology and BioImaging, Ludwig-Maximilians-Universität München, Munich 81377, Germany; Science for Life Laboratory, Department of Medical Biochemistry and Biophysics, Karolinska Institutet 17165 Stockholm, Sweden, Ming Wai Lau Centre for Reparative Medicine, Stockholm Node, Karolinska Institutet 17177 Stockholm, Sweden; Department of Proteomics and Signal Transduction, Max-Planck Institute of Biochemistry, Martinsried 82152, Germany; Faculty of Biology, Anthropology and Human Genomics, Ludwig-Maximilians-Universität München 82152, Planegg-Martinsried, Germany; Institute of Functional Epigenetics (IFE), Helmholtz Zentrum München, 85764 Neuherberg, Germany; Institute of Functional Epigenetics (IFE), Helmholtz Zentrum München, 85764 Neuherberg, Germany; Faculty of Biology and Center for Molecular Biosystems (BioSysM), Human Biology and BioImaging, Ludwig-Maximilians-Universität München, Munich 81377, Germany; Science for Life Laboratory, Department of Medical Biochemistry and Biophysics, Karolinska Institutet 17165 Stockholm, Sweden, Ming Wai Lau Centre for Reparative Medicine, Stockholm Node, Karolinska Institutet 17177 Stockholm, Sweden; Faculty of Biology and Center for Molecular Biosystems (BioSysM), Human Biology and BioImaging, Ludwig-Maximilians-Universität München, Munich 81377, Germany

## Abstract

DNA methylation (5-methylcytosine (5mC)) is critical for genome stability and transcriptional regulation in mammals. The discovery that ten-eleven translocation (TET) proteins catalyze the oxidation of 5mC to 5-hydroxymethylcytosine (5hmC), 5-formylcytosine (5fC), and 5-carboxylcytosine (5caC) revolutionized our perspective on the complexity and regulation of DNA modifications. However, to what extent the regulatory functions of TET1 can be attributed to its catalytic activity remains unclear. Here, we use genome engineering and quantitative multi-omics approaches to dissect the precise catalytic vs. non-catalytic functions of TET1 in murine embryonic stem cells (mESCs). Our study identifies TET1 as an essential interaction hub for multiple chromatin modifying complexes and a global regulator of histone modifications. Strikingly, we find that the majority of transcriptional regulation depends on non-catalytic functions of TET1. In particular, we show that TET1 is critical for the establishment of H3K9me3 and H4K20me3 at endogenous retroviral elements (ERVs) and their silencing that is independent of its canonical role in DNA demethylation. Furthermore, we provide evidence that this repression of ERVs depends on the interaction between TET1 and SIN3A. In summary, we demonstrate that the non-catalytic functions of TET1 are critical for regulation of gene expression and the silencing of endogenous retroviruses in mESCs.

## INTRODUCTION

DNA methylation is essential for the regulation of gene expression and genome stability in mammals (1). During development, methylated cytosine (5-methylcytosine (5mC)) serves as an epigenetic modification that prevents illegitimate cell fate decisions and contributes to coordination of the step-wise exit of pluripotency (2). The genome-wide landscape of 5mC is established during development by the de novo DNA methyltransferases DNMT3A and DNMT3B and maintained through subsequent cell divisions by the DNA methyltransferase DNMT1. The global 5mC patterns can be altered by the inhibition of maintenance DNA methylation and/or via the action of the Ten-eleven Translocation (TET) family of dioxygenases (3). The three mammalian homologs, TET1, TET2, and TET3 share a conserved C-terminal dioxygenase domain, which can catalyze the stepwise oxidation from 5mC to 5-hydroxymethylcytosine (5hmC), 5-formylcytosine (5fC), and 5-carboxylcytosine (5caC) (4–7). These oxidized cytosine derivatives have been described as intermediates of passive and active DNA demethylation (6,8–10), yet may also represent stable epigenetic marks on their own (11,12).

TET1 and TET3 possess a CXXC-type zinc finger domain that promotes their targeting to CpG-rich sequences, whereas TET2 associates with IDAX, an independent CXXC domain-containing protein (13). The expression of TET proteins is highly dynamic during mouse preimplantation development. TET3 is strongly expressed in oocytes and zygotes followed by rapid depletion over the following cleavage stages, while TET1 and TET2 expression increase up to the blastocyst stage (14–16). In murine embryonic stem cells (mESCs), TET1 and TET2 are the main TET proteins expressed, whereas TET3 is present at very low to undetectable levels (17). Loss of all TET proteins is incompatible with normal mammalian development (18–21), as evidenced by the failure of TET-deficient mice to develop beyond gastrulation (20,21). In comparison, single TET mutants exhibit less severe yet distinct phenotypes, suggesting that each enzyme can partially compensate for loss of the other (22–24).

TET proteins demethylate regulatory regions including promoters, enhancers and distal regulatory elements (25). For instance, R-loop-dependent demethylation by TET1 is critical for transcriptional activation of the *Tcf21* promoter (26) and active DNA demethylation mediated by TET1 and TET2 has been demonstrated to facilitate somatic cell reprogramming (27). Furthermore, TET-catalytic activity restricts Polycomb domain boundaries to the promoters of developmentally regulated genes (28). In general, active DNA demethylation by TET1 as well as TET2 is responsible for maintaining the distinctive global DNA hypomethylation signature of naive mESCs, albeit indirectly via the locus-specific demethylation and transcriptional activation of *Dppa3* (29). Beyond this, it has become increasingly clear that TET proteins also regulate transcription independently of their catalytic activity. For example, the phenotype of full-length TET1 knockout (KO) mice differs from that of mice lacking the TET1 catalytic domain (23). Furthermore, TET1 mainly suppresses gene expression independent of its DNA demethylase activity in adipocytes (Villivalam *et al.*, 2020). Similarly, TET2 can activate gene expression independent of its catalytic activity via the direct interaction with the O-linked *N*-acetylglucosamine (O-GlcNAc) transferase (OGT) (30).

TET1 binds through its CXXC domain, both active and bivalent promoters and can act as either a transcriptional repressor or activator depending on the associated chromatin modifying complexes (13). At this, TET1 interacts with several protein complexes including Polycomb Repressive Complex 2 (PRC2) and the SIN3A histone deacetylase (SIN3A/HDAC) complex to regulate transcription (31–33). Several early studies demonstrated that TET1 accumulates at PRC2 targets and promotes the recruitment of the histone 3 lysine 27 trimethylation (H3K27me3)-depositing enzyme EZH2 to these sites (31–34). In addition, TET1 is also described to associate with SIN3A/HDAC, OGT, the histone acetyltransferase MOF, and chromatin remodeler MBD3/NuRD (32,35–37). These findings suggest that TET1 can regulate gene expression by coordinating chromatin modifying complexes.

In addition to gene regulation, TET1 has also been implicated in the repression of transposable elements (TEs) (38). In vertebrates, TEs are highly decorated by DNA methylation, which is essential for genomic stability (39–43). Counterintuitively, in mESCs young non-long terminal repeat (non-LTR) LINE-1 (L1) elements are highly decorated with 5hmC and maintained in a hypomethylated state by TET1, while their repression is mediated by SIN3A in a TET1-dependent manner (38). Furthermore, LTR-containing endogenous retroviruses (ERVs) were described to be specifically upregulated in TET triple KO (TKO) mESCs potentially due to loss of TRIM28 (also known as KAP1) binding (25). Besides DNA methylation, retrotransposons are repressed by the establishment of histone 3 lysine 9 trimethylation (H3K9me3) and histone 4 lysine 20 trimethylation (H4K20me3) (44–46). However, it is unclear how TET1-SIN3A is involved in the silencing machinery, repressing L1 elements. Furthermore, it is an open question if TET1-SIN3A might also regulate the activity of LTR retrotransposons, such as ERVs.

Taken together, these findings suggest that TET1 can mediate transcriptional regulation in a catalytically independent manner. However, the underlying molecular mechanisms as well as the extent of TET1’s non-catalytic functions remain poorly understood.

Here, we systematically dissected the non-catalytic role of TET1 in mESCs. We used genome engineering and a quantitative multi-omics approach to compare a TET1 KO with a catalytically inactive TET1 mESC line. In particular, we find that (i) a large proportion of transcriptional changes are independent of TET1-mediated DNA demethylation; (ii) TET1 associates with different chromatin modifiers and is important for the establishment of specific histone modifications, namely H3K27me3, pan histone 4 lysine 5 + 8 + 12 + 16 acetylation (pH4Kac) and H4K20me3 and (iii) that loss of the TET1 protein but not its catalytic activity causes a specific loss of H3K9me3 at ERV1, ERVK and ERVL elements. Finally, we highlight that the interplay between TET1 and SIN3A is a main driver of ERV repression. Our results demonstrate that TET1 has a pivotal non-catalytic role in regulating gene expression and ERV silencing in mESCs.

## MATERIALS AND METHODS

### Cell culture

The generation of Tet1 KO (clone H9) and Tet1 CM (clone D7) mESC lines was described previously (17,29).

Mouse ESCs were cultured in ‘Serum LIF’ conditions and as independent replicates for 6 days prior to experiments. Here the cells were maintained on 0.2% gelatin-coated dishes in Dulbecco's modified Eagle's medium (Sigma) supplemented with 16% fetal bovine serum (FBS, Sigma), 0.1 mM ß-mercaptoethanol (Invitrogen), 2 mM l-glutamine (Sigma), 1× MEM Non-essential amino acids (Sigma), 100 U/ml penicillin, 100 μg/ml streptomycin (Sigma), homemade recombinant LIF tested for efficient self-renewal maintenance.

For the generation of piggybac doxycycline inducible cell lines, mESCs were cultured in ‘Serum LIF 2i media’. Those were the same conditions as described above, but supplemented with 2i (1 μM PD032591 and 3 μM CHIR99021 (Axon Medchem, Netherlands)).

All cell lines were regularly tested for Mycoplasma contamination by PCR.

### Piggybac constructs and cell line generation

The piggybac dox inducible TET1 (#102421) and TET1 CM (#102422) vector constructs were obtained from addgene (23). To generate the TET1 piggybac donor vector carrying a mutation at the Sin3a interaction domain (SID) (47), two overlapping PCR fragments were amplified.

Primers:

Sin3a_PsyI_FWD: 5’ gtccatggactgcagtagacgtggtcatggggaagaagagc 3’

Sin3a_NheI_REV: 5’ ttactatactctatagctagctgctcttgcttcttctgatc 3’

Sin3a_SID_FWD: 5’ caagtggtagccatagaagccGCCactcagGCCtcagaag 3’

Sin3a_SID_REV: 5’ cttctgaGGCctgagtGGCggcttctatggctaccacttg 3’

The resulting DNA fragments were cloned into the TET1 or TET1 CM piggybac vector digested with PsyI and NheI (Thermo Fisher Scientific) using a Gibson Cloning Kit (NEB).

To generate stable mESC lines carrying doxycycline-inducible forms of *Tet1, Tet1CM* or *Tet1 Sin3a mut.*, Tet1 KO mES cells were seeded at 0.5 mio mESCs in a 6-well plate and transfected with 1.5 μg of the pPB-tetO(hCMV1)-HA-Tet1mHxD(201R2)-IV (#102422, addgene) or pPB-tetO(hCMV1)-HA-Tet1(201R2)-IV (#102421, addgene) or pPB-tetO(hCMV1)-HA-Tet1Sin3a(201R2)-IV plasmid, 0.5 μg of the PiggyBac transposase vector (#PB200PA-1System, Biosciences) and 0.5 μg of the pPB-CAG-rtTA-IRES-Hygro (#102423, addgene) plasmid using Lipofectamine 3000 (Thermo Fisher Scientific) according to manufacturer's instructions. Two days after transfection, cells were plated at 10% confluency into a p100 plate and selected with Hygromycin (125 μg/ml) for 5–6 days. To enrich positive clones, cells were induced with doxycycline (1 μg/ml) for 24 h then sorted with flow cytometry on thresholded levels of mVenus expression. The mVenus fluorophore is under the control of the same promoter as Tet1 via an IRES sequence and therefore a fluorescent readout of successful induction. To ensure a stable pool the cell lines were sorted twice for mVenus expression. Post sorting, cells were plated back into media without doxycycline for 7 days before commencing experiments.

### Western blot

Western blots for TET1 rescue and HP1β were performed as described previously (48) using monoclonal antibody rat anti-TET1 5D6 (1:10) (49), rabbit anti-HP1β (1:1000, 10478, abcam), rabbit anti-HP1β (1:1000, 8676, Cell Signaling) and polyclonal mouse anti-Tubulin (1:2500; T9026, Sigma-Aldrich) as loading control. Briefly, 1 million cells were collected and washed with ice-cold PBS (D8537, Sigma-Aldrich). The cells were lysed in 75 μl ice-cold RIPA buffer (50 mM TRIS/HCl pH 8.0, 150 mM NaCl, 0.1% UltraPure™ SDS Solution (24730020, Invitrogen), 0.5% sodium deoxycholate detergent, 1% Triton X-100; freshly add 1× cOmplete™ EDTA-free Protease Inhibitor Cocktail (04693132001, Roche), 2 mM PMSF, 0.1 U/μl Benzonase), mixed with 25 μl 4× Laemmli and boiled for 10 min at 95°C. Samples were separated by 8% (TET1) and 10% (HP1β) SDS-Page Mini-Protean system (Bio-Rad) and transferred to a nitrocellulose membrane (0,2 μM) using wet transfer (Bio-Rad). After blocking (1h, 5% milk in PBS-Tween), the blots were probed with the before mentioned primary antibodies and the corresponding secondary antibodies goat anti-rat (1:5000; 112-035-068, Jackson ImmunoResearch), goat anti-rabbit (1:5000, 170-6515, Bio-Rad) and goat anti-mouse (1:5000; A9044, Sigma-Aldrich) conjugated to horseradish peroxidase (HRP) and visualized using an ECL detection kit (Thermo Scientific Pierce).

### MINUTE-ChIP

The quantitative multiplexed ChIP experiments were conducted as previously described (50). In short, three cell lines (WT J1, Tet1 KO H9 and Tet1 CM D7) were cultured as independent quadruplicates, cell pellets of 2 mio cells were lysed in Lysis Buffer and digested with 6 U/μl Micrococcal nuclease for 10 min at 37ºC. T7-adapters with 6 bp unique molecular identifying (UMI) sequences and 8bp sample barcodes were ligated to the chromatin fragments for 2 h at 23ºC and subsequently for 16 h at 16ºC. The twelve samples were thereafter pooled together and 2 mio cell equivalents of digested and barcoded chromatin was used for immunoprecipitation using antibodies for the histone marks H3K4me3 (04-745, Millipore), H3K27me3 (07-449, Millipore), H3K27me1 (61015, Active Motif), H4K20me3 (07-463, Millipore), H4K20me1 (ab9051, Abcam), pH4Kac (06-598, Sigma) and H3K9me3 (39161, Active Motif). The antibodies were coupled to SureBeads Protein A (1614013, Bio-Rad) and Protein G (1614023, Bio-Rad) magnetic beads and the immunoprecipitation was conducted for 4 h at 4ºC with rotation, followed by quick washes using RIPA and LiCl buffers. The immunoprecipitated chromatin was eluted from the beads and subjected to Proteinase K for 1 h at 63ºC. A sample consisting of 0.2 mio cell equivalent from the pooled lysates was also subjected to Proteinase K digestion as input for later normalization purposes. The digested DNA was cleaned up using AMPureXP SPRI beads (A63881, Beckman Coulter). The barcoded DNA fragments were in vitro transcribed for 16 h at 37ºC followed by DNase digestion for 15 min at 37ºC and purified using Silane beads (37002D, Thermo Fisher Scientific). RA3 RNA adapters were ligated to the transcripts for 2 h at 25ºC followed by reverse transcription to cDNA using a paired end primer. The cDNA was cleaned up using AMPureXP SPRI beads. 150 ng of cDNA was used for library PCR using a different barcoded primer for each sample. Finally, the libraries were diluted to 4 nM and combined for sequencing using Illumina sequencing.

### MINUTE-ChIP analysis

We conducted the MINUTE-ChIP data analysis as previously described (51). The bioinformatic pipeline for MINUTE-ChIP data analysis is available at github (https://github.com/NBISweden/minute).

#### Preparation of FASTQ files

Sequencing was performed using 50:8:34 cycles (Read1:Index1:Read2) Illumina bcl2fastq was used to demultiplex paired-end sequencing reads by 8nt index1 read (PCR barcode). NextSeq lanes were merged into single fastq files, creating the primary fastq files. Read1 starts with 6nt UMI and 8nt barcode in the format NNNNNNABCDEFGH.

#### Primary analysis

MINUTE-ChIP multiplexed FASTQ files were processed using minute, a data processing pipeline implemented in Snakemake (52). In order to ensure reproducibility, a conda environment was set. Source code and configuration are available on GitHub: https://github.com/NBISweden/minute. Main steps performed are described below.

#### Adaptor removal

Read pairs matching parts of the adaptor sequence (SBS3 or T7 promoter) in either read1 or read2 were removed using cutadapt v3.2 (53).

#### Demultiplexing and deduplication

Reads were demultiplexed using cutadapt v3.2 allowing only one mismatch per barcode. Demultiplexed reads were written into sample-specific fastq files used for subsequent mapping and GEO submission.

#### Mapping

Sample-specific paired fastq files were mapped to the mouse genome (mm10) using bowtie2 (v2.3.5.1) with –fast parameter. Alignments were processed into sorted BAM files with samtools (v1.10). Pooled BAM files were generated from replicates using samtools.

#### Deduplication

Duplicate reads are marked using UMI-sensitive deduplication tool je-suite (v2.0.RC) (https://github.com/gbcs-embl/Je/). Read pairs are marked as duplicates if their read1 (first-in-pair) sequences have the same UMI (allowing for 1 mismatch) and map to the same location in the genome. Blacklisted regions were then removed from BAM files using BEDTools (v2.29.2).

#### Generation of coverage tracks and quantitative scaling

Input coverage tracks with 1bp resolution in BigWig format were generated from BAM files using deepTools (v3.5.0) bamCoverage and scaled to a reads-per-genome- coverage of one (1xRPGC, also referred to as ‘1× normalization’). ChIP coverage tracks were generated from BAM files using deepTools (v3.5.0) bamCoverage. Quantitative scaling of the ChIP-Seq tracks amongst conditions within each pool was based on their Input-Normalized Mapped Read Count (INRC). INRC was calculated by dividing the number of unique mm10-mapped reads by the respective number of Input reads: #mapped[ChIP]/#mapped[Input]. This essentially corrected for an uneven representation of barcodes in the Input and we previously demonstrated that the INRC is proportional to the amount of epitope present in each condition (50). Wildtype mESC (replicates combined) were chosen as the reference condition, which was scaled to 1x coverage (also termed Reads per Genome Coverage, RPGC). All other conditions were scaled relative to the reference using the ratio of INRCs multiplied by the scaling factor determined for 1x normalization of the reference: (#mapped[ChIP]/#mapped[Input])/(#mapped[ChIP_Reference]/#mapped[Input_Reference]) × scaling factor.

#### Quality control

FastQC was run on all FASTQ files to assess general sequencing quality.

Picard (v2.24.1) was used to determine insert size distribution, duplication rate, estimated library size. Mapping stats were generated from BAM files using samtools (v1.10) idxstats and flagstat commands. Final reports with all the statistics generated throughout the pipeline execution are gathered with MultiQC (54).

### ChIP analysis of published data sets

We analysed published ChIP-seq reads of TET1 (34), SIN3A (55), SETDB1 (56) and H3K9ac (57) of WT mESC cultured in SL medium. Reads were aligned to the mouse genome (mm10) with Bowtie (v.1.2.2) with parameters ‘-a -m 3 -n 3 –best –strata’. Subsequent ChIP–seq analysis was carried out on data of merged replicates. Peak calling and signal pile up was performed using MACS2 callpeak (58) with the parameters ‘–extsize 150’ for ChIP, ‘–extsize 220–nomodel -B –nolambda’ for all samples. Reads mapping to Repeats (defined by RepeatMasker mm10) were extracted using custom R scripts.

### Enzymatic methylome sequencing (EM-seq)

Three cell lines (WT J1, Tet1 KO H9 and Tet1 CM D7) were cultured as independent triplicates. The genomic DNA was isolated using the QIAamp DNA Mini Kit (QIAGEN). DNA concentration was measured using Nanodrop (NanoPhotometer NP80, Implen). The gDNA was then diluted to 10 ng/μl in 200 μl TE buffer. To control the conversion efficiency 0.01 ng pUC19 methylated DNA and 0.2 ng unmethylated lambda DNA were added. The DNA was sheared into 350–400 bp fragments using the Bioruptor Plus sonication device (Diagenode) (30 s on/off, 20 cycles). Bioanalyzer (Agilent) was used to control for the shearing efficiency. For library preparation 200 ng of the sheared DNA were used. The final EM-seq library preparation was performed according to the manufacturer's instructions (New England Biolabs).

### EM-seq processing and analysis

The EM-seq library was a paired end sequencing run, 2 × 150 bp (Novogene). Raw reads were first trimmed using Trim Galore (v.0.3.1). Alignments were carried out to the mouse genome (mm10) using bsmap (v.2.90) using the parameters ‘-s 12 -v 10 -r 2 -I 1’. CpG-methylation calls were extracted from the mapping output using bsmaps methratio.py. Analysis was restricted to CpG with a coverage >10. methylKit (59) was used to identify differentially methylated regions between the respective contrasts for the following genomic features: (i) all 1-kb tiles (containing a minimum of three CpGs) detected by EM-seq; (ii) repeats (defined by RepeatMasker mm10); (iii) gene promoters (defined as gene start sites −2 kb/+2 kb) and (iv) gene bodies (defined as longest isoform per gene) and CpG islands (as defined by (60)). Differentially methylated regions were identified as regions with *P* <0.05 and a difference in methylation means between two groups >20%. DNA methylation browser track figures were created using IGV (v2.9.2).

### Relative quantification of histone post translational modification abundances using LC-MS/MS

Histones were acid extracted as described previously (61). In brief, mESCs were lysed in 10× cell pellet volume of ice-cold hypotonic lysis buffer (15 mM Tris–-HCl (pH 7.5), 60 mM KCl, 11 mM CaCl2, 5 mM NaCl, 5 mM MgCl2, 250 mM sucrose, 1 mM dithiothreitol, 10 mM sodium butyrate) supplemented with 0.1% NP-40 on ice for 5 min. Nuclei were pelleted by centrifugation (1000g, 2 min, 4^○^C) and washed twice in ice-cold hypotonic lysis buffer w/o NP-40. Nuclei were resuspended in 5× nuclei pellet volumes of ice-cold 0.2 M sulfuric acid and mixed on a rotation wheel for 120 min at 4°C. Insolubilized nuclear debris was pelleted by centrifugation (16 000g, 10 min, 4°C). Supernatant was transferred to a fresh low-protein binding Eppendorf tube and histone proteins were precipitated by adding ice-cold trichloroacetic acid (TCA) to the final concentration of 20% (v/v) followed by 60 min incubation on ice. Precipitated histone proteins were pelleted by centrifugation (16 000g, 10 min, 4°C), washed 3 times with acetone (–20°C) and resuspended in MS grade water.

Extracted histones were prepared for LC–MS/MS analysis using hybrid chemical derivatization method as described previously (62). In brief, 4 μg aliquots of purified histones were diluted with MS grade water to a total volume of 18 μl and buffered to pH 8.5 by addition of 2 μl of 1 M triethylammonium bicarbonate buffer (TEAB). Propionic anhydride was mixed with MS grade water in a ratio of 1:100 and 2 μl of the anhydride-mixture was added immediately to the histone sample, with vortexing, and the resulting mixture was incubated for 5 min at room temperature. The reaction was quenched by adding 2 μl of 80 mm hydroxylamine followed by 20 min incubation at room temperature. Tryptic digestion was performed overnight with 0.5 μg trypsin per sample at 37^○^C. A 1% v/v solution of phenyl isocyanate (PIC) in acetonitrile was freshly prepared and 6 μl added to each sample and incubated for 60 min at 37°C. Samples were acidified by adding trifluoroacetic acid (TFA) to the final concentration of 1%. Peptides were de-salted with C18 spin columns (Pierce™) following the manufacture protocol. Peptides were eluted from C18 spin columns with 70% acetonitrile, partially dried in a speedvac and resuspended in 30 μl 0.1% TFA.

The resulting peptide mixtures were analyzed using nano-flow liquid chromatography–tandem mass spectrometry (LC–MS/MS) on a Q-Exactive HF mass spectrometer coupled to an Ultimate 3000 nano-UPLC (Ultimate 3000, Dionex, Sunnyvale, CA) in data-dependant acquisition (DDA) mode. ∼300 ng peptide aliquot was used per one sample per one injection. Peptides were loaded automatically on a trap column (300 μm inner diameter × 5 mm, Acclaim PepMap100 C18, 5 μm, 100 Å; LC Packings, Sunnyvale, USA) prior to C18 reversed phase chromatography on the analytical column (nanoEase MZ HSS T3 Column, 100 Å, 1.8 μm, 75 μm × 250 mm; Waters, Milford, USA). Peptides were separated at flow rate of 0.250 μl per minute by a linear gradient from 1% buffer B (0.1% (v/v) formic acid, 98% (v/v) acetonitrile) to 25% buffer B over 40 min followed by a linear gradient to 40% B in 20 min, then to 85% B in 5 min. After 5 min at 85% buffer B, the gradient was reduced to 1% buffer B over 2 min and then allowed to equilibrate for 8 min. Full mass range spectra were at 60 000 resolution (at *m*/*z* 400), and product ions spectra were collected in a ‘top 15’ data-dependent scan cycle at 15 000 resolution.

RAW MS data were analyzed using EpiProfile 2.0 software (63). The reported relative abundances of histone modifications were validated by manual re-quantification using an open-source Skyline software.

### Cell growth and morphology analysis

The time evolution of cell growth and cell morphology was determined using the PHIO Cellwatcher (www.phio.de). WT J1, Tet1 KO and Tet1 CM mESCs lines were cultured in Serum LIF media as described. The Cellwatcher was placed inside the incubator and images with a large field of view of 10 mm² were automatically recorded every 30 min. The cell proliferation and morphology data were gained with PHIO’s automatic AI-based analysis platform and were accessed through PHIO’s data dashboard www.phio-cells.com.

For cell counting, WT J1, Tet1 KO and Tet1 CM mESCs lines were seeded in 6-well plates at densities of 0.35 mio mESCs/well in five replicates. The cells were collected and counted after 24 and 48 h using an automated cell counter (Countstar BioTech).

### RNA-seq library

For RNA-seq, three different cell lines (WT J1, Tet1 KO H9, Tet1 CM D7) were cultured as independent quadruplicates. RNA was isolated using the NucleoSpin Triprep Kit (Machery-Nagel) according to the manufacturer's instructions. Isolated total RNA was normalised and subjected to RNA sequencing using a version of the prime-seq method (64). This method is based on the single cell RNA-seq method mcSCRB-seq (65) and is a three prime counting method that includes a sample specific barcode sequence and unique molecular identifiers (UMI) for accurate quantification of gene expression. Here we used the Nextera XT Kit (Illumina) for sequencing library preparation as described in the mcSCRB-seq protocol (65). Illumina paired end sequencing was performed on an HiSeq 1500 instrument for the first two experiments and on a NextSeq 1000 instrument for the third experiment. The first read was 16–28 bases long and covered the sample barcode and UMI, the second read was 50–109 bases long and read the cDNA fragment. Raw data was demultiplexed using deML (66), adapters and poly A tails were trimmed using cutadapt (53) and further preprocessed using the zUMIs pipeline (67) with STAR (68). Reads were mapped to the mouse genome (mm10) with either Ensembl annotation for the first experiment (GRCm38 release 102) or Gencode annotation (v M25) for the later experiments.

### RNA-seq processing and analysis

RNA-seq libraries were processed and mapped to the mouse genome (mm10) using the zUMIs pipeline (67). UMI count tables were filtered for low counts using HTSFilter (69). Differential expression analysis was performed in R using DESeq2 (70) and genes with an adjusted *P* <0.05 and an LFC >abs(1) were considered to be differentially expressed. Differential expression analysis over transposable elements was performed using TEtranscript (71).

### Immunofluorescence staining

For immunostaining, mESCs were grown on coverslips coated with Geltrex (Life Technologies), thereby allowing better visualization during microscopic analysis. All steps during immunostaining were performed at room temperature. Coverslips were rinsed two times with PBS (pH 7.4; 140 mM NaCl, 2.7 mM KCl, 6.5 mM Na_2_HPO_4_, 1.5 mM KH_2_PO_4_) prewarmed to 37°C, cells fixed for 10 min with 4% paraformaldehyde (pH 7.0; prepared from paraformaldehyde powder (Merck) by heating in PBS up to 60°C; stored at –20°C), washed three times by dipping in PBST (PBS, 0.01% Tween20), permeabilized for 5 min in PBS supplemented with 0.5% Triton X-100, and washed two times by dipping in PBS. Primary and secondary antibodies were diluted in blocking solution (PBST, 4% BSA). Coverslips were incubated with primary and secondary antibody solutions (PBST, 4% BSA) in dark humid chambers for 1 h and washed three times by dipping in PBST after primary and secondary antibodies. For DNA counterstaining, coverslips were incubated 6 min in PBST containing a final concentration of 2 μg/ml DAPI (Sigma-Aldrich) and washed three times for 10 min with PBST. Coverslips were mounted in antifade medium (Vectashield, Vector Laboratories) and sealed with colorless nail polish.

Following primary antibodies were used: polyclonal rabbit anti-HP1β (1:300; 10478, abcam), monoclonal mouse anti-HP1α (1:100, 05-689, Sigma-Aldrich), monoclonal mouse anti-HP1γ (1:100, MA3-054, Invitrogen) and monoclonal rat anti-TET1 (1:10; 5D6). Following secondary antibodies were used: polyclonal donkey anti-rabbit Alexa 488 (1:500; 711-547-003, Dianova), polyclonal donkey anti-rat 488 (1:500, A-21208, Life technologies), polyclonal donkey anti-rabbit Alexa 647 (1:500, A-21244, ThermoFisher Scientific), polyclonal donkey anti-mouse Alexa 647 (1:500, A-31571, Invitrogen).

### Immunofluorescence imaging and analysis

Images were acquired on the Leica TCS SP8 X using 63× glycerol immersion objective and high-content screening Operetta microscope using a 20× objective. DAPI or fluorophores were excited with 405, 488 or 594 nm laser lines. Within each experiment, cells were imaged using the same settings on the microscope (camera exposure time, laser power and gain) to compare signal intensities between cell lines.

Images were analyzed using Fiji software (ImageJ 1.51j) for SP8 images and Harmony software package for Operetta images.

The coefficient of variance (CV) of the respective fluorescent signal was calculated as follows: (standard deviation/mean) × 100. The mean fluorescence and standard deviation of the fluorescence signal was acquired and calculated with the Operetta microscope and Harmony software package. To calculate the CV of the KO + TET1 and KO + TET1 SIN3A mut. rescue experiments, we used a TET1 antibody staining to identify cells with TET1 expression. The cells were separated into TET1 positive (488 nm mean intensity > 1500) and TET1 negative (488 nm mean intensity < 1500) and the CV calculated of the respective population.

### Mass spectrometry-based proteomic analysis of chromatin immunoprecipitated samples

Chromatin immunoprecipitation coupled to Mass Spectrometry (ChIP-MS) of TET1 was performed in triplicates for WT and TET1 KO mESCs under Serum LIF condition. For the pulldown a direct TET1 antibody (09-872-I, Sigma-Aldrich) was employed. ChIP-MS was performed as described previously, but without MNase digestion (72). Briefly, for each replicate a 15 cm cell culture dish was cultured for 2 days and 15 mio cells were crosslinked by 1% paraformaldehyde. Cells were lysed by the IP buffer (1.7% Triton X-100, 100 mM NaCl, 50 mM Tris–HCl pH 8.0, 5 mM EDTA pH 8.0, 0.3% SDS and freshly added 1x protease inhibitor cocktail) by pipetting and resting for 10 min on ice. Chromatin was sheared by sonication for 15 min in a Bioruptor Plus (30 s on/off cycles, Diagenode). Shearing efficiency was checked after overnight reverse crosslinking and proteinase K digestion of samples on a 1% agarose gel. Protein concentrations were estimated by BCA assay (Thermo) and samples were diluted to 1.3 mg/ml in 1 ml. 1.7 μg of the antibody was added to each replicate and samples were incubated O/N at 4°C under constant rotation. The next day magnetic protein A/G beads (20 μl slurry volume/sample, Sigma) were added to each sample to wash out unspecific interactors. After two low salt (50 mM HEPES pH 7.5, 140 mM NaCl, 1% Triton X-100), one high salt (50 mM HEPES pH 7.5, 500 mM NaCl, 1% Triton X-100) and two TBS washes, proteins were incubated in 2 mM DTT and subsequently 40 mM CAA (both diluted in 2 M Urea and 50 mM Tris–HCl pH 7.5). Then proteins were on-bead digested by Trypsin (20 μg/ml) O/N at 25°C. The next day, protease activity was stopped by 1% TFA and peptides were cleaned-up on Stage Tips consisting of three layers of C18 material (Empore) (73). After elution from Stage Tips peptides were speedvac dried and resuspended in 20 μl of A* buffer (0.1% TFA and 2% acetonitrile). Peptide concentrations were estimated by nanodrop measurements at 280 nm.

300 ng of each peptide solution was analyzed on a quadrupole Orbitrap mass spectrometer (Orbitrap Exploris™ 480, Thermo Fisher Scientific) after nanoflow liquid chromatography on an in-house packed 50 cm column (ReproSil-Pur C18-AQ 1.9 μM resin, Dr Maisch GmbH) coupled to an Easy-nLC 1200 (Thermo Fisher Scientific) over a linear acetonitrile gradient for 120 min. Data-dependent acquisition was employed and thereby the most abundant 12 peptides were selected for MS/MS scans. The target value for full scan MS spectra was set to 3 × 10^6^ and the resolution was at 60 000. The *m*/*z* range was adjusted to 400–1650 *m*/*z* and the maximum injection time was limited to 20 ms.

Subsequent data analysis of raw MS files was first accomplished by the MaxQuant software package (version 1.6.0.7) (74). Protein sequences were acquired over the Uniprot database (reviewed and unreviewed, version 2020) as a FASTA file. The MaxQuant analysis comprised the ‘Match between runs’ option, a false discovery rate for both peptides (minimum length of 7 amino acids) and proteins of 1% and determination of proteins amounts by the MaxLFQ algorithm (75). Downstream analysis was then performed with the Perseus software package (version 1.6.0.9). A two-sided Student's *t*-test of the log_2_ transformed LFQ intensities was performed to obtain significantly enriched proteins. By definition, a permutation-based false discovery rate of 5% and a fold change cut-off of log_2_ = 1 was applied.

## RESULTS

### TET1 regulates gene expression mainly independent of its catalytic activity in mESCs

**Figure 1.**
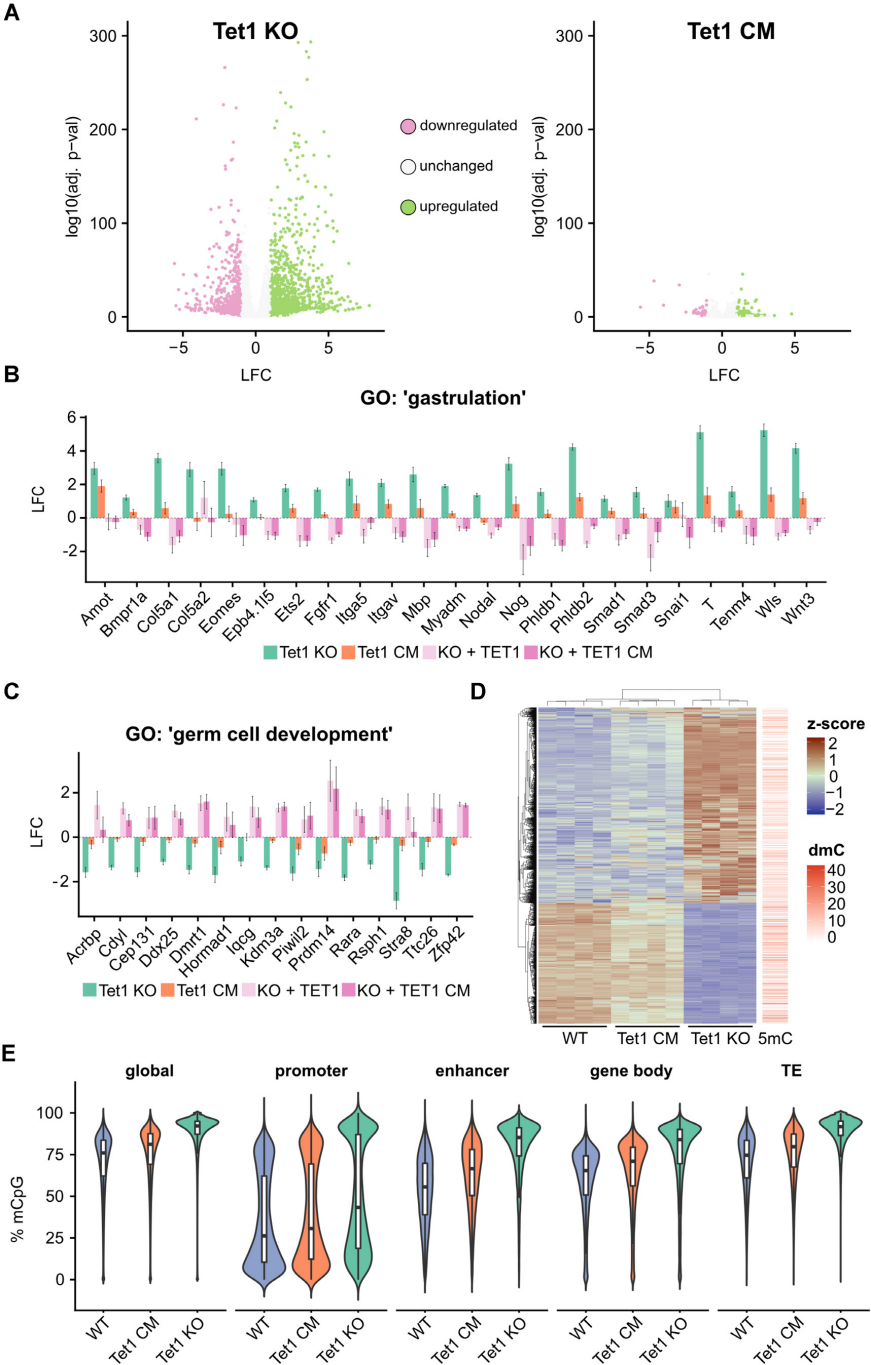
TET1 regulates gene expression mainly independent of its catalytic activity. (**A**) Volcano plots illustrating the transcriptional changes (log_2_-fold change, LFC) of Tet1 knockout (Tet1 KO) and catalytic mutant (Tet1 CM) mESCs relative to WT mESCs as assessed by RNA-seq. Green dots: Upregulated genes (Tet1 KO = 1250; Tet1 CM = 91). Violet dots: Downregulated genes (Tet1 KO = 770; Tet1 CM = 39). Grey dots: Unchanged expression. The threshold for significant changes was applied for an adjusted *P*-value <0.05 and LFC <–1 or >1 (*n* = 4 independent replicates). (**B**) Expression of selected genes from the GO cluster ‘gastrulation’, depicting the LFC of Tet1 KO and Tet1 CM relative to WT mESCs and Tet1 KO mESCs re-expressing TET1 or TET1 CM relative to Tet1 KO mESCs. (**C**) same analysis as in (B) depicted for genes in the GO term ‘germ cell development’ (*n* = 3 independent replicates). (**D**) Heat map of the hierarchical clustering of the RNA-seq expression *z*-scores and promoter DNA methylation in Tet1 KO mESCs significantly up- or downregulated genes. Promoter DNA methylation was assessed by enzymatic methylome sequencing (EM-seq, *n* = 3 independent replicates). Red bars indicate the delta DNA methylation (dmC, Tet1 KO – WT)) at the corresponding promoter. (**E**) Violin plots showing the percentage of methylated CpG dinucleotides globally, at promoters, enhancers, gene bodies and transposable elements (TE) in WT, Tet1 KO and Tet1 CM mESCs determined by EM-seq.

To dissect the catalytic and non-catalytic contributions of TET1, we used our previously described *Tet1* knockout (Tet1 KO) and *Tet1* catalytic mutant (Tet1 CM) mESCs (17,29). All cell lines were cultured in standard mESC media containing serum and leukemia inhibitory factor LIF (SL). We observed a striking difference in growth and morphology among wildtype (WT), Tet1 KO and Tet1 CM cells. Compared with WT and Tet1 CM cells, Tet1 KO mESC colonies exhibited a much flatter and less rounded morphology, a classical morphological hallmark of reduced pluripotency and spontaneous differentiation (Supplementary Figure 1A, B). While both Tet1 KO and Tet1 CM showed impaired cell growth, only Tet1 KO cells were altered in shape and size (Supplementary Figure 1A, B). To determine the transcriptional consequences of TET1 inactivation compared with total loss of TET1 proteins, we performed bulk RNA-seq (prime-seq (64)) on Tet1 KO, Tet1 CM, and WT mESCs. Differential gene expression analysis between WT and each of the TET1 mutant cell lines revealed that loss and catalytic inactivation of TET1 resulted in transcriptional activation as well as repression (Figure 1A), in line with TET1’s dual role in transcriptional regulation (33). Strikingly, however, we found in Tet1 KO mESCs ∼5 times more genes (2020) to be differentially expressed than in Tet1 CM mESCs (459). This small subset of genes deregulated in Tet1 CM mESCs was almost entirely composed of genes also deregulated in Tet1 KO mESCs (Supplementary Figure 2A), strongly suggesting that these are catalytically-dependent TET1 targets. While these catalytically-dependent genes exhibited the same directionality of expression changes (up- or downregulation) in both Tet1 KOs and Tet1 CMs, the extent of deregulation in terms of fold-change was more severe in Tet1 KO mESCs (Supplementary Figure 2B). This discrepancy in comparison to Tet1 CM mESCs implies that these genes are subject to synergistic catalytic and non-catalytic regulation by TET1. Next, we performed a Gene Set enrichment analysis to investigate whether genes controlled by TET1 cluster into functional groups. We detected several significantly deregulated gene sets with enriched Gene Ontology (GO) terms in the Tet1 KO mESCs, yet no encriched gene sets in the Tet1 CM mESCs (Supplementary Table 1). In line with our observation of a differentiated cell morphology upon TET1 loss, we found several developmental GO terms such as ‘gastrulation’, ‘embryonic organ development’, and ‘cell differentiation’ enriched among significantly upregulated genes in Tet1 KO mESCs. In contrast, significantly downregulated genes in Tet1 KOs were associated with naive pluripotency GO terms such as ‘germ cell development’, ‘response to leukemia inhibitory factor’, and ‘spermatogenesis’ (Supplementary Table 1). These findings indicate that TET1 is important for maintaining the balance between pluripotency and lineage commitment.

To further investigate whether these changes in gene expression are dependent or independent of TET1’s catalytic activity, we performed two rescue experiments. In particular, we used PiggyBac-mediated transposition to stably express TET1 or TET1 CM in Tet1 KO mESCs upon induction with doxycycline (Supplementary Figure 2C) (23). We then performed bulk RNA-seq (prime-seq (64)) to study the global effect on the transcriptome upon re-expression of TET1 or TET1 CM. In contrast to reintroducing TET1, TET1 CM cannot stimulate active DNA demethylation and hence cannot rescue genome-wide DNA modification levels (23). However re-expression of both TET1 or TET1 CM resulted in the repression of developmental markers upregulated in Tet KO cells such as genes involved in gastrulation (e.g. *Ets2*, *Mbp* and *Nog*, Figure 1B, Supplementary Figure 2D). Similarly, genes downregulated in Tet1 KO cells such as those involved in germ cell development (e.g. *Zfp42* and *Prdm14*) were upregulated after re-expression of either TET1 or TET1 CM (Figure 1C, Supplementary Figure 2D). Taken together, these results are consistent with previous findings (32,33,76) and reveal that loss of TET1 results in the upregulation of developmental genes as well as the downregulation of naive pluripotency markers. Remarkably, we find that TET1 controls these genes largely independently of its catalytic activity.

Finally, we asked whether the transcriptional dysregulation in TET1 mutant ESCs might be attributable to changes in DNA methylation. To address this question we performed enzymatic methylome sequencing (EM-seq, Supplementary Table 2). Strikingly, the loss of TET1 resulted in widespread promoter hypermethylation (Supplementary Figure 2E). However, we found that, in the majority of cases, increased promoter methylation was not accompanied by changes in gene expression, in line with previous studies (23,77,78) (Figure 1D, Supplementary Figure 2E). Only a small cluster of genes were found to be both downregulated and exhibit promoter hypermethylation in Tet1 KO as well as Tet1 CM mESCs, suggesting that there are relatively few bona fide catalytic targets of TET1 (Supplementary Figure 2E and F). The majority of studies have reported hypermethylation (17,23,32,79,80) while some have shown hypomethylation in Tet1 KO mESCs (81,82). Overall, we observed genome-wide hypermethylation in Tet1 KO mESCs, which was less pronounced in Tet1 CM mESCs (Figure 1E). We detected an increase in DNA methylation at promoter, enhancer, gene bodies and TEs in Tet1 KO and Tet1 CM compared to WT mESCs. DNA methylation gains were broadly correlated between Tet1 KO and Tet1 CM, with Tet1 KO showing a larger effect size (Figure 1E, Supplementary Figure 2G). Collectively, we found that TET1 predominantly regulates gene expression independently of its catalytic activity with only a small subset of genes depending on promoter demethylation by TET1.

### Loss of TET1 alters the chromatin modification landscape

**Figure 2.**
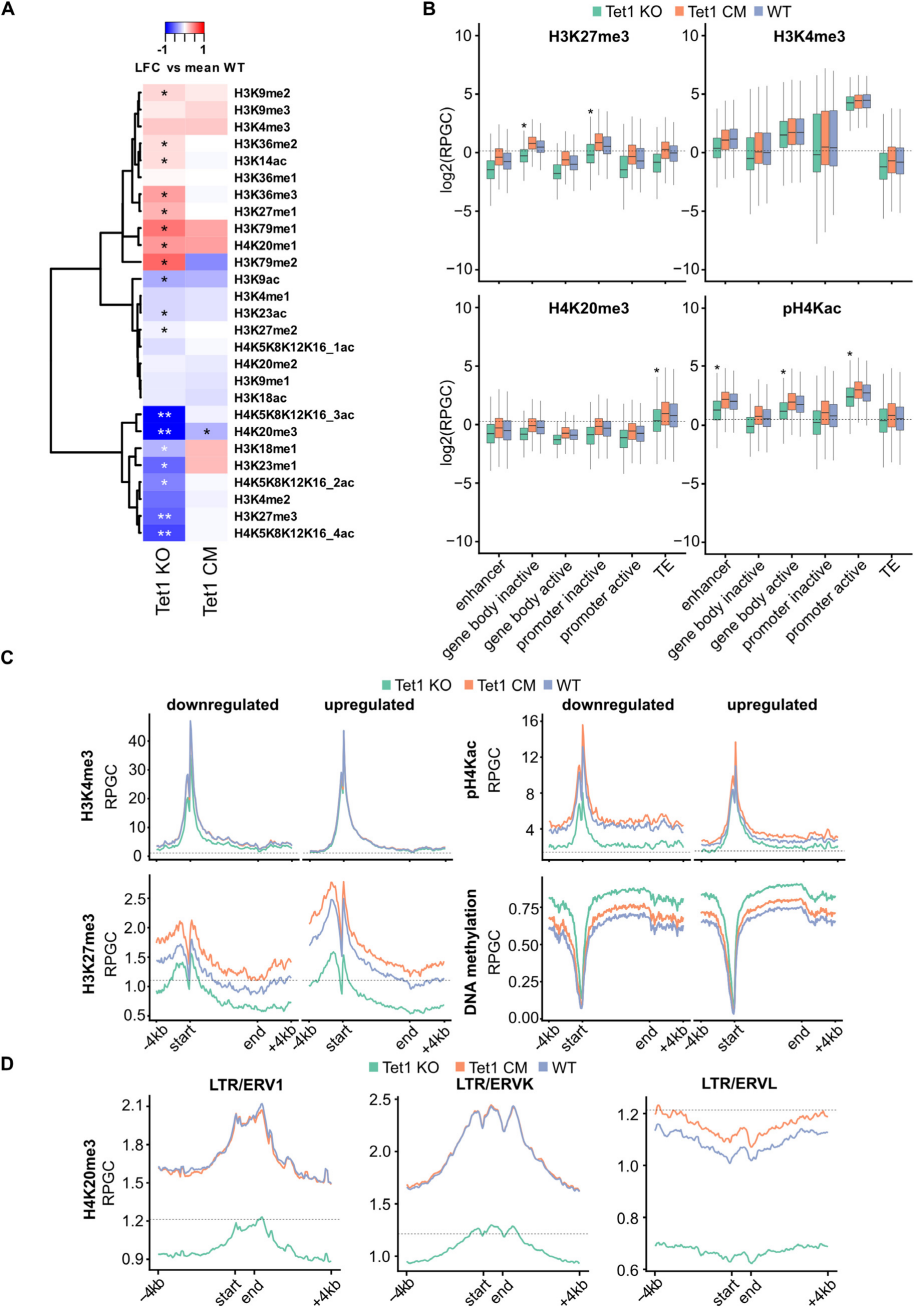
Tet1 KO mESCs display a reduction in histone marks. (**A**) Heatmap depicting hierarchical clustering of individual histone post-translational modification abundances. Calculated is the log_2_-fold change (LFC) relative to the mean abundances in WT mESCs. LC-MS/MS quantification of Tet1 KO, Tet1 CM, and WT mESCs (*n* = 3 independent replicates). Each row represents distinct histone modification states and the color gradient indicates the LFC. Significant changes (adjusted *P*-value < 0.05 and < 0.01) in the Tet1 KO and CM relative to WT mESCs are marked with * and **, respectively. (B–D) The y-axis indicates reads per genomic content (RPGC). The dotted line indicates the genome average RPGC of the respective histone signal (*n* = 4 independent replicates). (**B**) Average quantitative MINUTE-ChIP signal displayed as boxplots of H3K27me3, H3K4me3, H4K20me3 and pH4Kac comparing Tet1 KO, Tet1 CM and WT at enhancer, gene body inactive/active, promoter inactive/active and transposable element (TE). Significant changes were marked with * (one-sided *t*-test, adjusted *P*-value <0.05, Tet1 KO relative to WT mESCs), see Supplementary Table 3 for a full list. Horizontal black lines within boxes represent median values, boxes indicate the lower and upper quartiles, and whiskers indicate the 1.5 interquartile range. (**C**) Average quantitative MINUTE-ChIP profiles of H3K4me3, H3K27me3, and pH4Kac and DNA methylation levels using EM-seq data in Tet1 KO, Tet1 CM and WT mESCs across gene bodies significantly down- or upregulated in Tet1 KO mESCs. Up- and downregulated genes were preselected for TET1 binding in WT mESCs. TET1 binding sites were identified using published ChIP-seq data of wild-type mESC cultured with the same medium conditions (34). (**D**) Average quantitative MINUTE-ChIP profiles of H4K20me3 across ERV1, ERVK and ERVL elements ±4 kb in Tet1 KO, Tet1 CM and WT mESCs.

To gain further insights into possible mechanisms by which TET1 regulates transcription independent of DNA demethylation, we asked if the loss of TET1 is accompanied by changes in the chromatin landscape. To this end, we compared the relative abundances of core histone modifications among Tet1 KO, Tet1 CM and WT mESCs using quantitative LC–MS/MS analysis. We observed a profound global reduction of H3K27me3, pH4Kac as well as H4K20me3 in Tet1 KO mESCs (Figure 2A, Supplementary Figure 3A). Conversely, the corresponding mono-methylation states H3K27me1 and H4K20me1 were significantly, but to a lower extent increased in Tet1 KO mESCs (Figure 2A). We also detected significant, albeit less pronounced changes of several other histone modifications such as H3K18me1, H3K23me1, H3K9ac and H3K14ac in Tet1 KO mESCs (Figure 2A, Supplementary Figure 3A). Similar to the transcriptomics data, these profound changes in histone modification levels were only observed in Tet1 KO cells with the exception of H4K20me3, which exhibited a modest downregulation in Tet1 CM cells (KO = 49% and CM = 18% reduction compared to WT) (Figure 2A, Supplementary Figure 3A). Notably, we observed a significant downregulation of the *EZH2* transcript level. However, in total these global reductions in histone modification levels in Tet1 KO mESCs cannot be explained by transcriptional deregulation of the responsible histone modifying enzyme complexes (Supplementary Figure 3B). Taken together, these results demonstrate that TET1 predominantly regulates global H3K27me3, pH4Kac and H4K20me3 histone modification states via catalytic-independent mechanisms.

To investigate how loss of TET1 affects the genomic distributions of H3K27me3, pH4Kac and H4K20me3, we acquired genome-wide histone modification profiles using the quantitative ChIP-Seq method MINUTE-ChIP (50). MINUTE-ChIP uses a barcoding and pooling approach to enable quantitative comparisons between samples. This allowed us to profile quadruplicates of WT, Tet1 CM and KO mESCs in the same pool. The global readcount analysis from these MINUTE-ChIP experiments confirmed the global trends observed by mass spectrometry, with Tet1 KO mESCs exhibiting significantly reduced levels of H3K27me3, pH4Kac and H4K20me3 (Supplementary Figure 4). Of note, in contrast to the LC-MS/MS data, global H4K20me3 levels were unchanged in the MINUTE-ChIP data from Tet1 CM mESCs.

Next, we focused our analysis on the distribution of H3K27me3, H3K4me3, pH4Kac and H4K20me3 across selected genomic elements including active promoters, inactive promoters, enhancers, gene bodies of active and inactive genes, and TEs (Figure 2B). For H3K27me3, pH4Kac and H4K20me3, we detected a strong reduction over all analyzed genomic elements in Tet1 KO mESCs, but only minimal reductions in H3K4me3. In general, most histone modifications such as H3K4me3, H3K27me3 and H4K20me3 exhibit well-defined patterns of enrichment over distinct genomic elements in WT mESCs (44). In line with prior reports, H3K4me3 was found at enhancers, active genes, and mainly at active promoters and, as in histone LC–MS/MS measurements, changed only subtly in Tet1 KO and Tet1 CM mESCs (Figure 2A and B). H3K27me3 was mainly enriched at inactive promoters and within inactive gene bodies, but significantly reduced upon TET1 loss (Figure 2B). Furthermore, pH4Kac was enriched at enhancers, active promoters, and within active gene bodies. At all three elements we observed a significant reduction in Tet1 KO mESCs (Figure 2B). We also found H4K20me3 to be enriched over TEs, but significantly reduced in Tet1 KO mESCs (Figure 2B). Additionally, we performed a chromatin-state discovery and genome annotation analysis with ChromHMM to investigate the enrichment of H3K4me3, H3K27me3, H3K27me1, pH4Kac, H4K20me3 and H4K20me1 at defined chromatin states. Amongst many smaller alterations, we detected a pronounced loss of H3K27me3 at poised promoters and a strong reduction of H4K20me3 at H3K9-marked heterochromatin (Supplementary Figure 5).

Next, we wondered whether the reduction of histone marks at promoters correlates with changes in gene expression and DNA methylation observed in Tet1 KO mESCs. We compared H3K4me3, H3K27me3, pH4Kac and DNA methylation levels over genes down- or upregulated in Tet1 KO mESCs. To narrow our focus on direct targets of TET1, we used published ChIP-seq data to preselect for genes bound by TET1 (34). We observed in Tet1 KO mESCs a reduction of H3K27me3 at upregulated genes, whereas at downregulated genes changes in H3K27me3 were less prominent (Figure 2C). H3K4me3 levels were unchanged at upregulated genes, but were slightly decreased at downregulated genes (Figure 2C). Furthermore, we detected a strong loss of pH4Kac at downregulated genes in Tet1 KO mESCs and almost no change at upregulated genes (Figure 2C). We asked if the changes in histone modification levels at up- and downregulated genes correspond to DNA hyper- or hypomethylation. We observed DNA hypermethylation at up- and downregulated genes in Tet1 KO mESCs and a similar but smaller increase in DNA methylation in Tet1 CM mESCs (Figure 2C). At multiple gastrulation and germ cell development markers, the loss of specific histone modifications correlated with expression changes observed in Tet1 KO mESCs. For instance, we detected a pronounced loss of H3K27me3 but only minor changes in H3K4me3 at the genomic locus of the upregulated gastrulation marker *Wnt3* in Tet1 KO mESCs. In contrast, the downregulated germ cell development marker *Zfp42* exhibited a clear loss of pH4Kac only in the Tet1 KO mESCs (Supplementary Figure 6A). In both cases we observed an increase of DNA methylation in Tet1 KO mESC at the promoter region and gene body (Supplementary Figure 6A). In summary, our data shows that the transcriptional deregulation observed in Tet1 KO mESCs cannot be attributed to changes in DNA methylation but rather global perturbation of histone modifications.

Since H4K20me3 was mainly enriched over TEs, we next analyzed whether H4K20me3 was specifically lost at distinct TE families in Tet1 KO mESCs. We detected a major loss of H4K20me3 at ERV1 and ERVK elements (Figure 2D, Supplementary Figure 6B). Additionally, we detected a less pronounced loss at L1 and ERVL elements (Figure 2D, Supplementary Figure 6B). TET1 seems to mainly regulate H4K20me3 levels at ERV1 and ERVK elements, raising the intriguing question how TET1 is involved in heterochromatin formation at these genetic elements. Collectively, we identify the non-catalytic role of TET1 to be a global regulator of H3K27me3, pH4Kac and H4K20me3 levels.

### TET1 associates with different chromatin modifiers and regulates ERV expression

**Figure 3.**
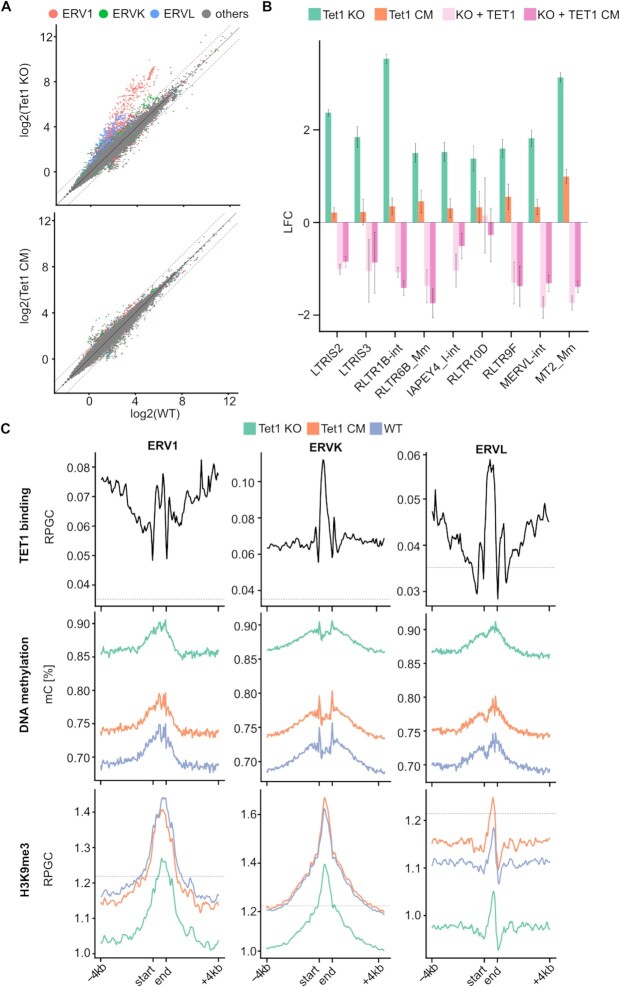
TET1 regulates H3K9me3 deposition and ERV silencing. (**A**) Scatter plot depicting log2 transformed counts of single TEs (transposable elements) comparing Tet1 KO versus WT and Tet1 CM versus WT. Red dots: ERV1, green dots: ERVK, blue dots: ERVL and grey dots: other TEs. Significantly upregulated ERV elements in Tet1 KO mESCs: ERV1 (*n* = 522), ERVK (*n* = 50), ERVL (*n* = 695). (**B**) LFC of differentially expressed ERVs in Tet1 KO relative to WT mESCs. Comparing ERV expression in Tet1 KO and Tet1 CM relative to WT mESCs and ERV expression when re-expressing TET1 or TET1 CM in Tet1 KO relative to Tet1 KO mESCs. LFC = log_2_ fold change (*n* = 3 independent replicates). (**C**) Average quantitative MINUTE-ChIP profiles of H3K9me3, ChIP profile of TET1 binding using published ChIP-seq data of mESC cultured under the same medium conditions (34) and percentage of DNA methylation using EM-seq data across ERV1, ERVK and ERVL elements ±4 kb (kilo base) comparing Tet1 KO, Tet1 CM and WT. For ChIP the y-axis shows reads per genomic content (RPGC). The dotted line indicates the genome average RPGC of the respective histone signal (*n* = 4 independent replicates).

We next asked if the dramatic drop in H4K20me3 at ERVs also correlates with changes in TE expression. In contrast to Tet1 CM mESCs, we identified in our RNA-seq data of Tet1 KO mESCs multiple TEs that were significantly upregulated (Figure 3A). In line with their loss of H4K20me3, we observed the strongest upregulation at ERV1 and ERVK elements (Figure 3A and B, Supplementary Figure 6B, 7). However, ERVL elements exhibited the greatest number of significantly upregulated ERVs in Tet1 KO mESCs (*n* = 695), compared to ERV1 (*n* = 522) and ERVK (*n* = 50) (Figure 3A). Furthermore, expression of exogenous TET1 or TET1 CM was able to reverse the ERV upregulation in Tet1 KO mESCs (Figure 3B), suggesting that ERVs are regulated independently of TET1’s catalytic activity.

Previously, TET1 binding was shown to strongly correlate with CpG density (32–34). In line with this observation, we found that ERV1 and ERVK elements in particular displayed a higher CpG density than expected by their GC content (Supplementary Figure 6C) and ERV elements with a higher observed over expected (O/E) CpG ratio were also more likely to be upregulated in Tet1 KO mESCs (Supplementary Figure 6C and D). Furthermore, using published TET1 ChIP-seq data (34) we found that TET1 was enriched at ERV1, ERVK and ERVL elements (Figure 3C). At the same time, all three ERV classes were hypermethylated in Tet1 KO mESCs. In the Tet1 CM mESC the increase in DNA methylation was significant, but less pronounced compared to Tet1 KO mESCs (Figure 3C). Our finding that DNA methylation is not sufficient to silence ERV elements in mESCs is in line with previous studies (83,84). Taken together, these findings suggest that TET1 binds ERV1, ERVK and ERVL elements due to their high CpG density and facilitates a repressive mechanism which is independent of DNA methylation and involves H4K20me3.

**Figure 4.**
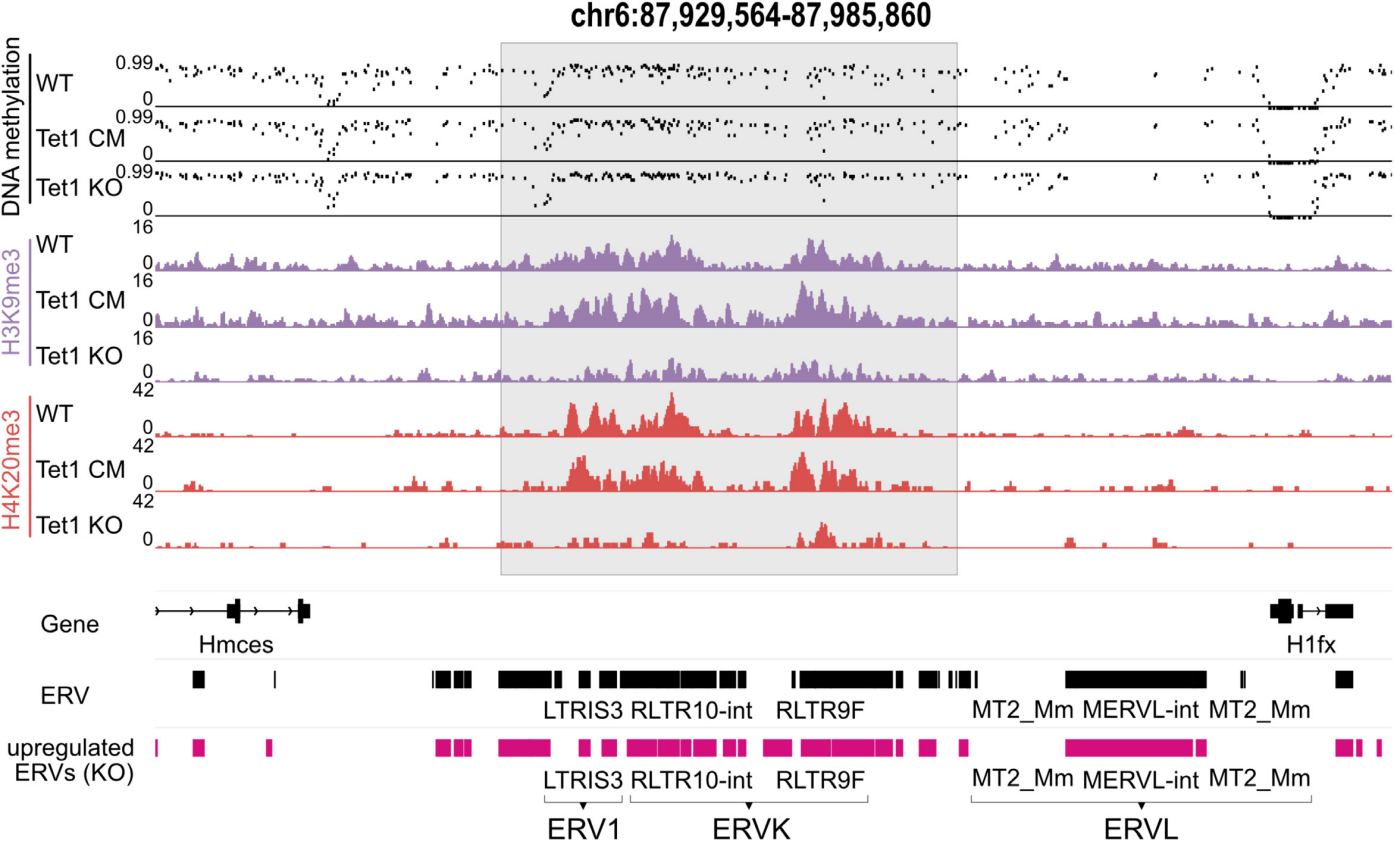
DNA methylation, H3K9me3 and H4K20me3 at upregulated ERVs in Tet1 KO mESCs. Representative genome browser tracks of EM-seq data, H3K9me3 and H4K20me3 ChIP in WT, Tet1 KO and Tet1 CM. Pink bars indicated the log fold change of ERVs upregulated in Tet1 KO cells. Individual upregulated ERVs are named and classified in ERV1, ERVK and ERVL. Regions with a gain of DNA methylation and loss of H3K9me3 and H4K20me3 in Tet1 KO cells are marked in grey.

The repression of TEs, especially of ERVs, relies on the cooperation of several epigenetic pathways. In particular, the establishment and maintenance of H3K9me3 is crucial for ERV1 and ERVK silencing (83). However, we did not detect a global loss of H3K9me3 in our histone LC–MS/MS measurements in Tet1 KO mESCs (Figure 2A). To investigate if H3K9me3 is specifically lost at ERVs in Tet1 KO mESCs, we exploited our quantitative MINUTE-ChIP approach. In accordance with the LC–MS/MS data, we did not observe a global reduction of H3K9me3 in Tet1 KO mESCs using quantitative ChIP-seq (Supplementary Figure 4 and 6E). Moreover, when all TEs were assessed as a single group, H3K9me3 levels appeared to be essentially unchanged in Tet1 KO mESCs. However, a more detailed analysis of individual TE families revealed a significant drop of H3K9me3 at ERV1, ERVK and ERVL in Tet1 KO mESCs (Figure 3C). We found that in Tet1 KO mESCs at specific ERV elements the loss of H3K9me3 and H4K20me3 co-occurs with an increase in DNA methylation and an upregulation of ERV elements (Figure 4). ERVL transcriptional activation correlates with the expression of the 2C marker *Zscan4* (85). In Tet1 KO mESCs, we detected a significant upregulation of the ERVL elements *MERVL-int* and *MT2_Mm* and the *Zscan4* cluster (Figure 3B, Supplementary Figure 6F). Interestingly, the activation of ERVL and *Zscan4* was significantly stronger in Tet1 KO compared to Tet1 CM mESCs and we detected a significant loss of both H3K9me3 and H4K20me3 at *MERVL-in*t and *MT2_Mm* (Supplementary Figure 7 and 8). In addition, we could rescue the *MERVL-int*, *MT2_Mm*, and *Zscan4* expression by reintroducing TET1 and TET1CM (Figure 3B and Supplementary Figure 6F). Previously, TET-mediated DNA demethylation was reported to regulate ERVL and *Zscan4* expression (23,25). In contrast, our data indicates a predominant non-catalytic role of TET1. Collectively, these findings describe a novel role of TET1 in ERV silencing independent of DNA demethylation. We demonstrate for the first time that TET1 is critical for H3K9me3 and H4K20me3 deposition and silencing of ERV1 and ERVK.

### The interplay between TET1 and SIN3A is crucial for ERV repression

**Figure 5.**
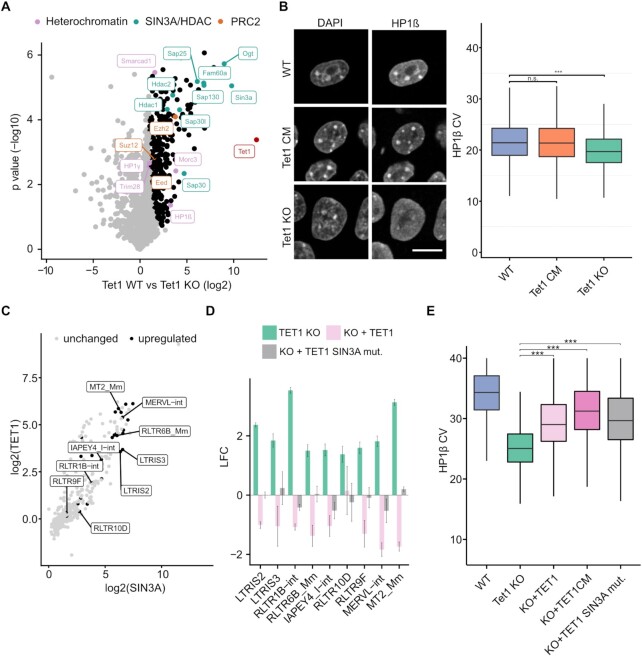
The TET1-SIN3A interaction is crucial for ERV regulation. (**A**) Volcano plot of TET1 ChIP-MS experiment in WT and Tet1 KO mESCs (*n* = 3 independent replicates). Black dots: significantly enriched after TET1 pulldown. Purple dots: Proteins associated with heterochromatin formation. Turquoise dots: Members of the SIN3A/HDAC complex. Orange dots: Core complex members of PRC2. Statistical significance is determined by performing a Student's *t*-test with a permutation-based false discovery rate of 0.05 and a cutoff of >1 of log_2_ transformed fold change. (**B**) Left: Immunofluorescence images of WT, Tet1 CM and Tet1 KO mESC stained for DAPI and HP1β. Scale bar = 10 μm. Images were taken using a confocal microscope. Right: Boxplots showing the coefficient of variation (CV) calculated from HP1β signal intensities, comparing WT (*n* = 27 588), Tet1 CM (*n* = 40 160), and Tet1 KO (*n* = 25 882). Images were taken using an Operetta microscope. ANOVA + Tukey's honestly significant difference post-hoc test: ****P < 0.0001. (**C**) Scatter plot comparing log2 transformed fold change enrichment of TET1 and SIN3A at transposable elements (TE) using published ChIP-seq data from mESCs cultured under the same conditions (34,55). Gray dots: unchanged expression of TE in Tet1 KO relative to WT mESCs. Black dots: upregulated TE in Tet1 KO relative to WT mESCs. (**D**) Expression of differentially expressed ERVs in Tet1 KO relative to WT mESCs as log_2_ transformed fold changes. Comparing ERV expression of Tet1 KO relative to WT mESCs and re-expressing TET1 or TET1 SIN3A mut. in Tet1 KO mESCs relative to Tet1 KO mESCs. (**E**) Boxplots depicting the coefficient of variation (CV) calculated from HP1β signal intensities comparing WT (*n* = 4617), Tet1 KO (*n* = 9334), KO + TET1 (*n* = 3757), KO + TET1CM (*n* = 1136) and KO + TET1 SIN3A mut. (*n* = 1885) TET1 and TET1 SIN3A mut. negative and positive cells. For the TET1 rescue cell lines, TET1 staining was used to select for TET1 positive (signal intensity > 1000) mESCs before the CV was calculated. ANOVA + Tukey's honestly significant difference post-hoc test: ****P < 0.0001. Horizontal black lines within boxes represent median values, boxes indicate the lower and upper quartiles, and whiskers indicate the 1.5 interquartile range. Representative confocal images of HP1β and TET1 stainings (Supplementary Figure 10).

Next, we aimed to investigate the underlying mechanism that regulates TET1-dependent silencing of ERV1, ERVK, and ERVL elements. Since we found that deposition of H3K9me3 and H4K20me3 is dependent on non-catalytic activities of TET1, we performed ChIP-MS on TET1 to identify interaction partners potentially involved in this process. Using this strategy, we identified a large number of different chromatin modifiers associating with TET1 (Figure 5A). In line with previous studies, we detected the core PRC2 complex (EED, SUZ12, EZH2) and many subunits of the SIN3A/HDAC complex (31,32). Strikingly, we also identified heterochromatin protein 1 (HP1) beta (HP1β, also known as CBX1), MORC3 and SMARCAD1 to be significantly enriched, and TRIM28 as well as HP1 gamma (HP1γ also known as CBX3) just below significance threshold (Figure 5A). Interestingly, these proteins were found to be associated with the formation of H3K9me3-marked heterochromatin in particular at ERVs (83,86–88).

A well-established pathway in ERV silencing is the binding of HP1 proteins to H3K9me3, recruiting SUV39H and SUV4-20H, and the subsequent spreading of H3K9me3 and H4K20me3 (89). To investigate if the ERV-specific loss of H3K9me3 might impact HP1β localization, we used immunofluorescence to examine the distribution of HP1 in Tet1 KO, Tet1 CM and WT mESCs. Intriguingly, HP1β became depleted from heterochromatic foci, i.e. chromocenters and exhibited an overall more homogenous distribution in the nucleus upon loss of TET1 protein but not upon loss of TET1 catalytic activity (Figure 5B). At the same time we observed only a minor reduction of HP1β at the transcript level and no obvious change on the protein level in Tet1 KO mESCs (Supplementary Figure 9A). To quantify our observation, we performed high-throughput microscopy and calculated the coefficient of variation (CV) of the HP1β signal, commonly used as a benchmark for fluorescence signal distribution (90,91). High CV values correspond to a heterogenous and lower CV values to a more homogenous signal distribution. While the HP1β signal in WT and Tet1 CM mESCs displayed similar CV values, we observed significantly lower CV values for HP1β in Tet1 KO mESCs (Figure 5B). In addition to HP1β, mammals possess two other paralogs of HP1, namely, HP1α and HP1γ. All three have overlapping, but distinct functions in heterochromatin formation (92,93). Therefore, we also analyzed the CV values of HP1α and HP1γ under the same conditions as for HP1β in WT, Tet1 KO and Tet1 CM mESCs. Compared with HP1β, the distribution of HP1α exhibited a more limited but still significant reduction in focal heterochromatin accumulation in Tet1 KO mESCs (Supplementary Figure 9B). In the case of HP1γ, the extent of this reduction in heterogeneity was even more severe in Tet1 KO mESCs (Supplementary Figure 9C). Although not as dramatic as Tet1 KO mESCs, we also observed significant decreases in the focal patterning of both HP1α and HP1γ in Tet1 CM mESCs (Supplementary Figure 9B, C). In summary, our data indicates that TET1 associates with heterochromatin proteins and might be a regulator of HP1 formation at heterochromatic regions.

It is well accepted that the turnover of histone acetylation is crucial for heterochromatin formation (94–97). Since we and others have found the SIN3A/HDAC complex to be among the most abundant interactors of TET1 (32,98,99), we investigated whether the TET1-SIN3A/HDAC interaction is involved in TET-mediated regulation of ERVs. To this end, we first assessed whether SIN3A occupies the same ERVs as TET1. We identified a considerable overlap between TET1 and SIN3A bound ERVs, many of which were also found to be upregulated in Tet1 KO mESCs (Figure 5C, Supplementary Figure 9D). Next, we asked whether the TET1 and SIN3A interaction is critical for the transcriptional regulation of these ERVs. To answer this question, we expressed a version of TET1 harbouring a mutation described to disrupt the interaction with SIN3A (TET1 SIN3A mut.) in Tet1 KO mESCs (Supplementary Figure 9E) (47). The two amino acids (L897 and L900) critical for the SIN3A interaction are not part of the catalytic domain of TET1. Intriguingly, ERV repression was restored by WT TET1, but not the TET1 SIN3A mut. (Figure 5D). Of note, we also identified a subset of genes where the TET1 SIN3A mut. rescues gene expression (e.g. *Esrrb*, *Lefty* and *Pvalb*), suggesting additional pathways independent of SIN3A (Supplementary Figure 9F).

**Figure 6.**
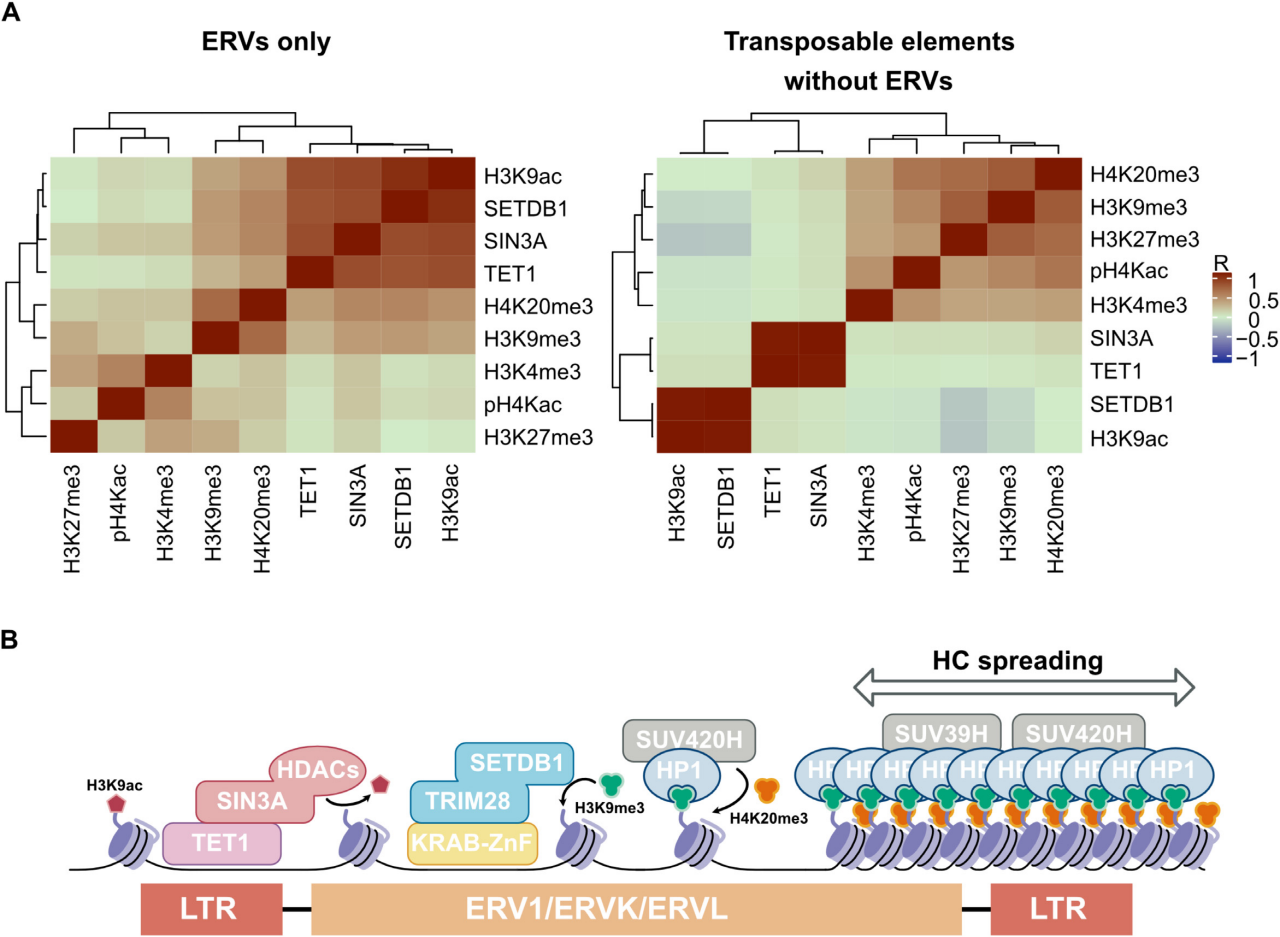
TET1-SIN3A/HDAC-mediated acetylation turnover might regulate H3K9me3/H4K20me3-mediated silencing of ERVs in mESCs. (**A**) Correlation matrix of ChIP-seq data of H3K9ac, SETDB1, SIN3A, TET1, H3K9me3, H4K20me3, pH4Kac, H3K4me3, and H3K27me3 at individual copies of only ERV elements (*n* = 258 668) or at individual copies of transposable elements (TEs) excluding ERVs (n = 757,079). The correlation coefficient (*R*) is indicated by a color gradient. (**B**) Model figure illustrating the proposed TET1-SIN3A/HDAC-mediated ERV1, ERVK and ERVL silencing mechanism. TET1 recruits the SIN3A/HDAC complex to ERV1, ERVK and ERVL elements. SIN3A/HDAC-mediated deacetylation of H3K9ac facilitates the recruitment of the KRAB-ZnF/TRIM28/SETDB1 silencing complex and the subsequent installation of the heterochromatin mark H3K9me3. HP1 proteins bind H3K9me3, recruit SUV39H and SUV4-20H for the establishment of H3K9me3 and H4K20me3 domains, ultimately causing heterochromatin (HC) spreading.

Finally, we asked whether reexpresing TET1 can restore HP1β localization. After re-expressing TET1, TET1CM and the TET1 SIN3A mut., we selected TET1 positive mESCs using a TET1 antibody staining and calculated the CV of the HP1β signal for WT, Tet1 KO and the three rescue cell lines. The re-expression of TET1, TET1CM and TET1 SIN3A mut. restored the HP1β localization to heterochromatic regions (Figure 5E, Supplementary Figure 10). Interestingly, the TET1 SIN3A mut. efficiently restored HP1β localization, but in contrast to WT TET1 did not silence ERV expression (Figure 5D). These findings are in line with the observation that HP1 proteins alone are not sufficient to silence ERVs in mESCs (100) and might suggest that the TET1-SIN3A mut. can still directly recruit HP1β to heterochromatin, but not silence ERV expression without SIN3A deacetylation activity. Deacetylation of the H3 tail is crucial for H3K9 methylation efficiency by SETDB1 (101). To investigate if H3K9ac, SETDB1, TET1 and SIN3A correlate at ERV1, ERVK and ERVL elements we used published ChIP-seq data (34,56,57) and our MINUTE-ChIP data of H3K9me3, H4K20me3, pH4Kac, H3K4me3 and H3K27me3. Interestingly, we found that H3K9ac, SETDB1, SIN3A and TET1 occupancy were highly correlated at ERV elements (Figure 6A). On the contrary, at all other TEs excluding ERVs, TET1 and SIN3A binding were not associated with SETDB1 and H3K9ac occupancy (Figure 6A). This might suggest that TET1-SIN3A are involved in deacetylation and the subsequent methylation of H3K9 via SETDB1 to control repression specifically of ERV elements. In summary, we identified TET1 as a key regulator of ERV expression in mESCs. Furthermore, our findings suggest that SIN3A is important for DNA demethylation independent regulation of ERVs by TET1.

## DISCUSSION

Whereas the role of TET1 in active DNA demethylation is well described (102), the non-catalytic functions of TET1 remain unclear. In contrast to earlier studies suggesting that TET1 KO mice are viable (18,22), a recent study reported that TET1 KO mice display severe gastrulation defects and are not viable after E9.5 (23). These discrepancies can be assigned to differences in the *Tet1* knockout targeting strategy. The viability of some *Tet1* KO strains seems to be the consequence of a hypomorphic deletion, which allows an N-terminal fragment of TET1 to be expressed. Importantly, this fragment does not contain the catalytic domain of TET1, suggesting TET1 to have key non-catalytic functions (23). Here, we aimed to systematically decipher those DNA demethylation independent functions of TET1 in mESCs.

In agreement with the current literature, our transcriptomics analysis revealed a deregulation of pluripotency and gastrulation markers in Tet1 KO mESCs (22,23,103,104). Interestingly, our rescue experiments, DNA methylation analysis and systematic comparison of Tet1 KO and Tet1 CM mESCs showed that the transcriptional changes can mainly be attributed to the non-catalytic functions of TET1. These findings are supported by a number of previous studies, suggesting a non-catalytic role of TET1 in mESCs, reprogramming or thermogenesis (23,27,32,105). While this manuscript was in the review process, another study demonstrated that TET1 regulates H3K27me3 in mESCs independent of its catalytic activity (106). In line with our observations, Chrysanthou *et al.* showed that TET1 regulates developmental genes together with PRC2 and SIN3A independent of its DNA demethylation activity. Further, non-catalytic functions of TET1 are critical for early development, while the catalytic functions gain importance in late gestation and postnatal development (106). To note, our RNA-seq and rescue data also shows some minor transcriptional effects in Tet1 CM mESCs at genes significantly deregulated in Tet1 KO mESCs. Therefore, in some cases the catalytic and non-catalytic functions of TET1 might cooperate to regulate transcription. These findings and several other studies highlight the relevance of TET1-dependent active DNA demethylation in different biological systems (102,107–110). Together, suggesting that TET1 catalytic functions are highly context-dependent. TET1 is also important for recruiting TET2 to chromatin (37). In mESCs and different biological settings, TET2 might be partially compensating for the catalytically inactive TET1. Those compensatory effects of TET2 or the blocking of CpG sites by the presence of catalytic inactive TET1 could explain the less pronounced hypermethylation in Tet1 CM mESCs observed in our EM-seq data. In line with this hypothesis, we and others recently proposed that TET1 and TET2 have coordinated roles in DNA demethylation (111). While active DNA demethylation in mESCs seems to have few transcriptional effects, TET catalytic functions in DNA demethylation or the oxidative derivatives 5hmC, 5fC and 5caC themselves are important during differentiation, gastrulation and in somatic cells. This hypothesis is supported by previous findings, demonstrating that TET-dependent active DNA demethylation at promoters of lineage factors is critical for their activation during lineage commitment, gastrulation and reprogramming (20,77,112).

The predominant non-catalytic role of TET1 in mESCs prompted us to further study the underlying mechanisms of TET1 regulating transcription. TET1 was previously shown to associate with the chromatin modifying complexes PRC2, SIN3A/HDAC, OGT, MBD3/NURD and MOF (31,32,35–37,98,99). Here, we used a LC–MS/MS approach to identify the global interplay of TET1 with different histone modifications. Whereas loss of TET1 was reported to result in a reduction of H3K27me3 at promoters (34,36), our data reveals a genome-wide reduction of this mark independent of TET1 catalytic activity. Additionally, we identified a global reduction of H4K20me3 as well as pH4Kac only in Tet1 KO and not in Tet1 CM mESCs. It has been suggested that TET1-dependent DNA demethylation facilitates other chromatin modifiers to bind and restructure chromatin in order to activate or repress transcription. In contrast, our data suggest that in mESCs active DNA demethylation by TET1 is not required for the proper regulation of chromatin states, as we did not detect global alterations of histone modifications in Tet1 CM. Further, changes in DNA methylation did in most cases not correlate with the observed gene expression and histone modifications changes, suggesting a DNA methylation independent mechanism in mESCs. Alternatively, TET1 might act as an interaction hub for chromatin modifiers and/or is important for the composition of different regulatory chromatin complexes.

Our data identifies TET1 as a novel interactor of the heterochromatin machinery and a regulator of ERV elements. We show that ERV1, ERVK and ERVL lose H3K9me3 and H4K20me3 in Tet1 KO mESCs. Furthermore, we find that TET1 associates with different proteins involved in heterochromatin formation. SMARCAD1 is a chromatin remodeler and was recently shown to regulate IAP elements (86), however we only observed a minor upregulation of most IAPs in Tet1 KO mESCs. Only recently, MORC3 was identified as a regulator of ERV elements and H3K9me3 (87). Among others, MORC3 regulates the LTRIS family, which we found significantly upregulated in Tet1 KO mESCs. To this end, future studies will be important to dissect a potential TET1-MORC3 interplay in ERV silencing.

In general, only little is known about the role of TET1 in ERV silencing. Previously, TET enzymes were proposed to regulate ERVL LTRs (25). ERVL expression is related to *Zscan4* expression and other markers of the 2 cell (2C) state (85). Interestingly, the *Zscan4* cluster was reported to be regulated by DNA demethylation (23). In contrast, our data indicates a more prominent upregulation in Tet1 KO mESC than in Tet1 CM mESCs. Furthermore, we could rescue the 2C markers when reexpressing TET1 CM in Tet1 KO mESCs. These findings indicate that *Zscan4* and MERVL regulation depend on both DNA demethylation and non-catalytic functions of TET1. In general, the finding that 2C markers are upregulated is contradictory to the concurrent upregulation of differentiation markers in Tet1 KO mESC. Serum LIF cultured mESCs exhibit a heterogeneous cell population and are known to include 2C-like cells (113). One explanation could be that the loss of TET1, besides mainly priming cells for differentiation, also promotes the expansion of the 2C-like cell subpopulation in Serum LIF mESC cultures.

Despite hypermethylation at ERVs in Tet1 KO mESCs, we could rescue normal ERV repression when reintroducing either TET1 or TET1 CM. Our finding that TET1 regulates ERV expression independently of its DNA demethylation function is in line with the observation that ERV silencing mediated by TRIM28 and SETDB1 is DNA methylation independent (83,84,114,115). In addition, non-LTR containing LINE1 elements are repressed independent of DNA methylation turnover, but by SIN3A in a TET1-dependent manner (38). To note, TRIM28/SETDB1 can also act synergistically with DNA methylation to silence IAP elements (88). One possible explanation for the simultaneous hypermethylation and activation of ERVs in Tet1 KO mESCs could be that 5mC-insensitive transcription factors are able to engage ERVs in the absence of TET1 (116).

Using immunofluorescence, we demonstrate for the first time that loss of TET1 leads to a displacement of HP1β, HP1γ, and HP1α from heterochromatin foci. Our data does not show that HP1 proteins are lost at ERVs in Tet1 KO mESCs. However, the loss of H3K9me3 and H4K20me3 at ERVs could explain the displacement of HP1 proteins from heterochromatic regions, prompting the question how TET1 influences the maintenance of heterochromatin in mESCs. The current model of heterochromatin formation proposes that site specific KRAB-Znf transcription factors recruit TRIM28 and its interaction partner SETDB1 to DNA. The latter installs H3K9me3, which is bound by HP1 and subsequently recruits SUV39H and SUV4-20H for spreading of H3K9me3 and H4K20me3 (89,117). We cannot completely rule out an indirect effect causing the loss of H3K9me3 and H4K20me3 in Tet1 KO mESC. However, our rescue experiments and quantitative ChIP data showing ERV silencing upon TET1 expression together with a specific loss of H3K9me3 at ERVs suggest that TET1 acts upstream of SETDB1. The loss of H3K9me3 in Tet1 KO mESCs could explain the delocalization of HP1β. Our interaction data and HP1β rescue experiments suggest that TET1 might also directly interact with HP1β independently of SIN3A and recruit HP1β directly to specific ERVs without inducing repression. This hypothesis would be in line with the finding that the deletion of HP1α, β, or γ alone does not lead to deregulation of ERV1 and ERVK, showing that TRIM28/SETDB1-mediated H3K9me3 deposition is sufficient for ERV silencing (100).

It is important to note that deacetylation and heterochromatin establishment are tightly connected (94,101,118–122). Furthermore, deacetylation of the H3 tail by SIN3A/HDAC is necessary for transcriptional repression and the loss of SIN3A causes a delocalization of HP1α (123) (101,121,124). Intriguingly, our TET1 ChIP-MS data identified a large number of the SIN3A/HDAC complex members as interactors. TET1 might be important for SIN3A/HDAC recruitment or complex composition, as SIN3A lacks any DNA-binding activity (125). Additionally, our rescue data strongly suggests that TET1 regulates ERVs in a SIN3A-dependent manner. Correlating binding of SETDB1, SIN3A, and TET1 and levels of H3K9ac, H3K9me3, and H4K20me3 revealed an overlap at ERV1, ERVK and ERVL elements, but not at other groups of TEs (Figure 6A). Therefore, we propose that the TET1-SIN3A/HDAC axis is crucial to control the constant acetylation turnover at ERV1, ERVK and ERVL, enabling the repression and installation of H3K9me3/H4K20me3 by TRIM28-SETDB1 (Figure 6B). We suggest that in mESC loss of TET1 interferes with correct placement and function of the SIN3A/HDAC complex at ERV elements. Subsequent accumulation of H3K9ac could interfere with TRIM28 or SETDB1 recruitment, resulting in a reduction of H3K9me3 at ERV1, ERVK and ERVL elements, displacement of HP1, and following loss of H4K20me3 (Figure 6B). It will be intriguing to further decipher the details of the underlying mechanism in the future.

Collectively, our results demonstrate that TET1 regulates gene expression independently of active DNA demethylation in mESCs. We provide novel insights into the mechanisms underlying TET1’s non-catalytic functions in transcriptional regulation, including identifying TET1 as a global regulator of histone modifications. Moreover, we show that TET1 associates with different proteins involved in heterochromatin formation to suppress the expression of ERV1, ERVK and ERVL elements. Finally, we provide evidence that the mechanism of TET1-mediated silencing of ERV1, ERVK and ERVL elements critically depends on the interaction between TET1 and SIN3A but not the catalytic activity of TET1. Our study reveals the importance of disentangling the non-catalytic and catalytic roles of TET enzymes in different biological contexts. This will be of particular relevance for furthering our understanding of *Tet* mutations and their molecular consequences in cancer and disease.

## DATA AVAILABILITY

EM-seq and RNA-seq data generated in this study is available at https://www.ebi.ac.uk/arrayexpress/ via the accession numbers E-MTAB-10933 and E-MTAB-10937, respectively. ChIP-seq data is available under the accession number GSE183465 at https://www.ncbi.nlm.nih.gov/geo/. The mass spectrometry proteomics data have been deposited to the ProteomeXchange Consortium via the PRIDE (126) partner repository with the dataset identifier PXD028566 and PXD028850.

## Supplementary Material

gkac642_Supplemental_Files
