# Supplementary material for: TET1 regulates gene expression and repression of endogenous retroviruses independent of DNA demethylation
Source: Nucleic Acids Res. 2022 Jul 29;50(15):8491–511. doi: 10.1093/nar/gkac642 (PMC9410877; doi:10.1093/nar/gkac642)
Supplement: gkac642_Supplemental_Files [file gkac642_supplemental_files.zip › Supplementary Information.pdf]

# Supplementary Information

## Supplementary Figure 1

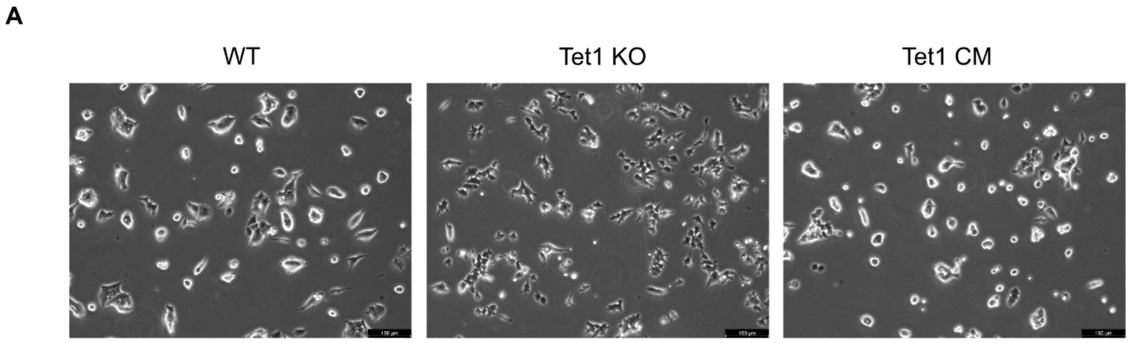


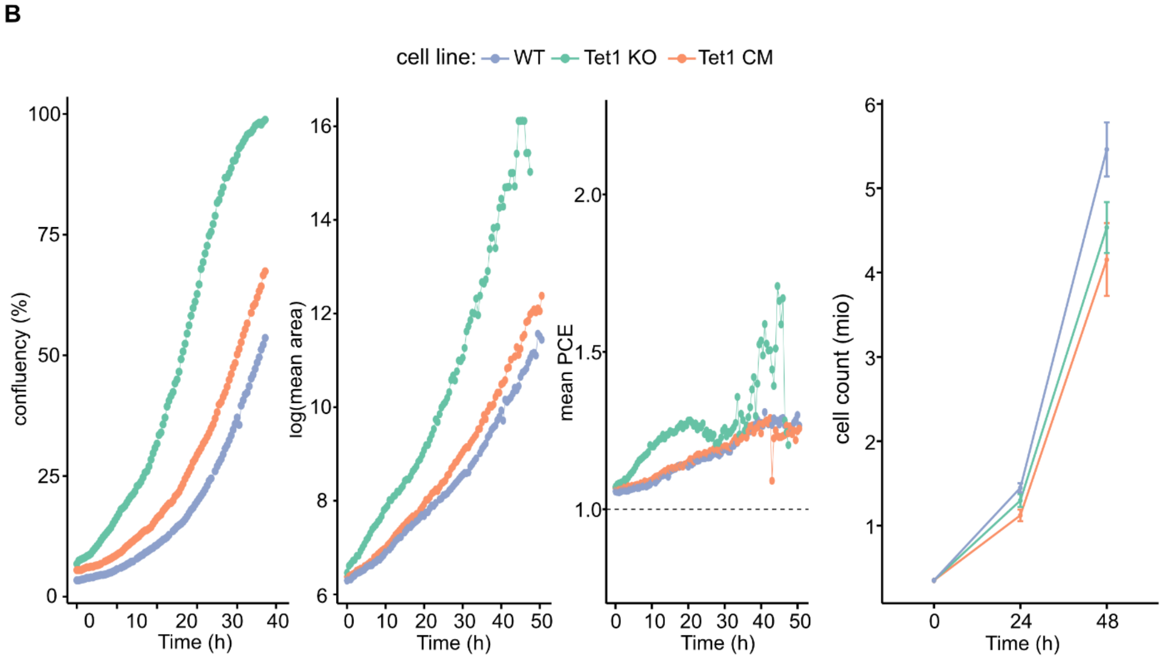


**Supplementary Figure 1. Tet1 KO mESCs exhibit growth and morphology differences compared to WT and Tet1 CM mESCs.** (A) Bright field images of WT, Tet1 CM, and Tet1 KO mESCs cultured in Serum LIF (SL). (B) Cell growth and morphology data collected using a PHIO Cellwatcher microscope depicting confluency (%), log mean area (µm), and mean perimeter circle equivalent (PCE) of mESC colonies/clusters over time (h). Cell counting was obtained using an automated cell counter (Countstar BioTech). Per cell line 0,35 mio mESCs were seeded and counted after 24h and 48h (n = 5). PCE is a measure for the roundness of a cell colonie/cluster, where the dotted line (PCE = 1) represents the perfect round circle. Data points represent Tet1 KO, Tet1 CM, and WT mESCs cultured in Serum LIF for one passage over 48h.

## Supplementary Figure 2

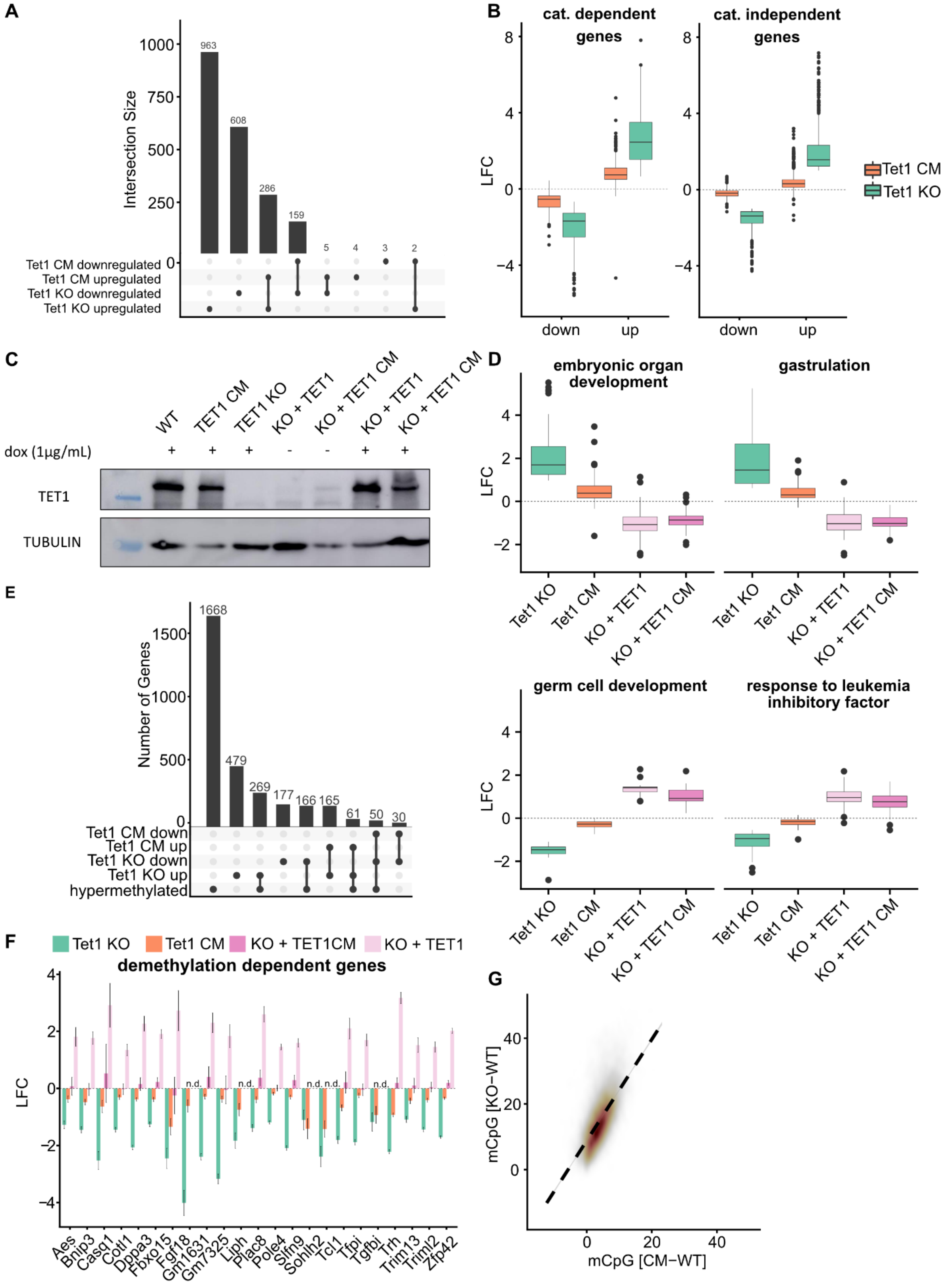


**Supplementary Figure 2. TET1-mediated transcriptional regulation in mESCs is predominantly independent of its catalytic activity.** (A) UpSet plot depicting the

overlap among genes classified as significantly down- or upregulated in Tet1 KO and/or Tet1 CM mESCs relative to WT in RNA-seq (n = 4 independent replicates). (B) Gene expression changes of TET1 targets. Left: gene expression changes of catalytic-dependent (cat. dependent) genes characterized by transcript levels significantly deregulated in Tet1 KO and Tet1 CM mESCs. Right: Gene expression changes of catalytic independent genes characterized by transcript levels only significantly deregulated in Tet1 KO, but not in Tet1 CM mESCs. (C) Western blot stained for TET1 and TUBULIN as loading control, showing TET1 levels in WT, Tet1 CM, Tet1 KO, and in the doxycycline (dox) inducible TET1 and TET1 CM rescue cell lines. Dox induction is indicated with (+) and absence of dox with (-). (D) Changes in GO cluster expression calculated as LFC comparing Tet1 KO and Tet1 CM relative to WT mESCs and the TET1 rescues KO + TET1 and KO + TET1 CM relative to Tet1 KO mESCs. The boxplots represent and are divided into the 4 different GO categories: ‘embryonic organ development’ ,‘gastrulation’, ‘germ cell development’, and ‘response to leukemia inhibitory factors’. (E) UpSet plot depicting the overlap between hypermethylated promoters in Tet1 KO mESCs assessed by enzymatic methylome sequencing (EM-seq, n = 3 independent replicates) and genes significantly downregulated or upregulated in Tet1 KO and Tet1 CM mESCs relative to WT. The respective number of genes is indicated on top of each bar. (F) Changes in transcript levels for 21 DNA demethylation-dependent genes harboring a hypermethylated promoter identified by EM-seq. Transcript levels in Tet1 KO and Tet1 CM relative to WT mESCs and in Tet1 KO mESCs re-expressing TET1 or TET1CM relative to Tet1 KO mESCs. n.d. = not detected. LFC = log2 fold changes. Horizontal black lines within boxes represent median values, boxes indicate the lower and upper quartiles, and whiskers indicate the 1.5 interquartile range. (G) Density plot of methylated CpG sites comparing Tet1 KO and Tet1 CM. The dashed line represents the linear correlation between the two data sets.

**Supplementary Figure 3**

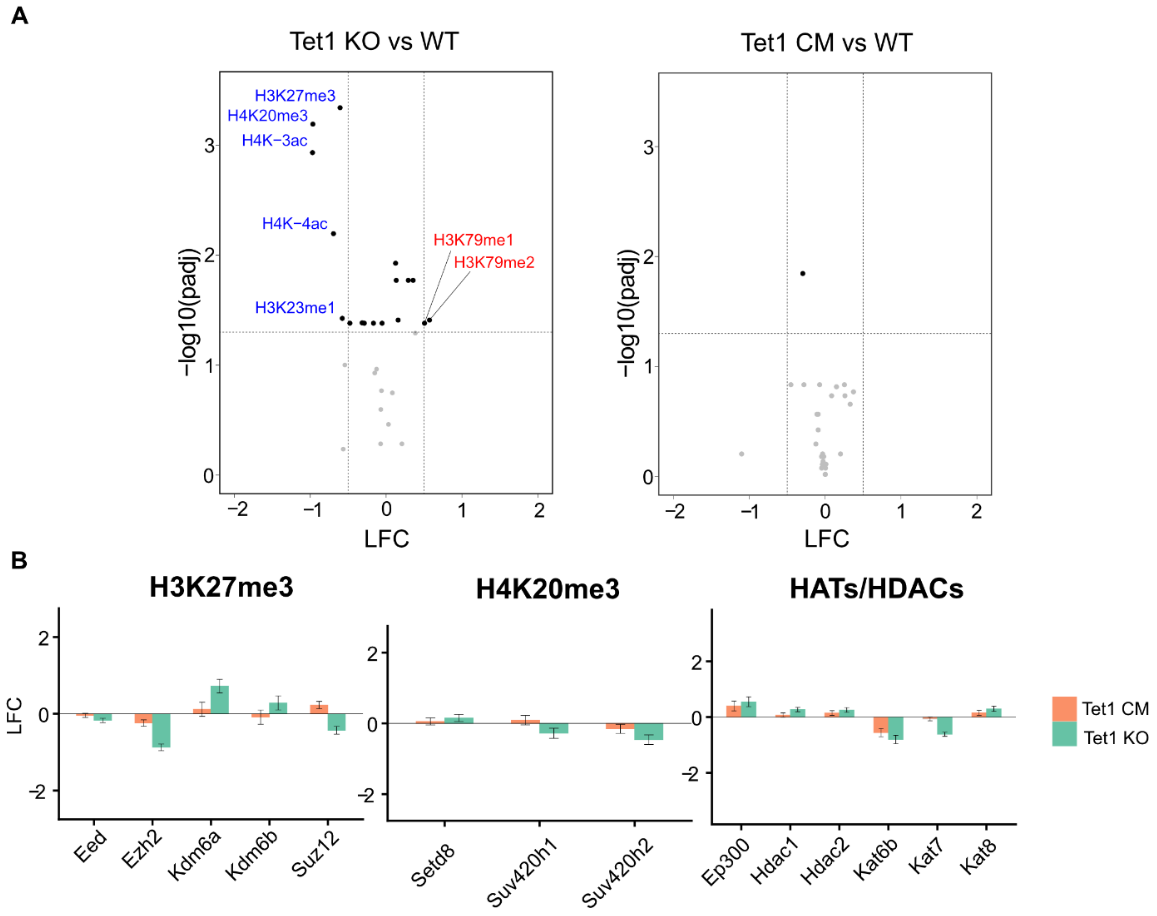


**Supplementary Figure 3. Loss of TET1 protein but not its catalytic activity results in genome-wide depletion of H3K27me3, H4K20me3, and pH4Kac.** (A) Volcano plots depicting log2 fold change (LFC) in relative abundances of individual histone modifications in Tet1 KO and Tet1 CM compared to WT mESCs. Significantly up- and downregulated modifications that show most prominent changes (|log2FC|> 0.5) are marked red and blue, respectively. (B) Changes (LFC of Tet1 KO or Tet1 CM relative to WT mESCs) in transcript levels of proteins involved in the deposition and/or removal of H3K27me3 (left), H4K20me3 (center), and histone acetylation (right). HAT/HDACs: histone acetyltransferases/histone deacetylases.

**Supplementary Figure S4**

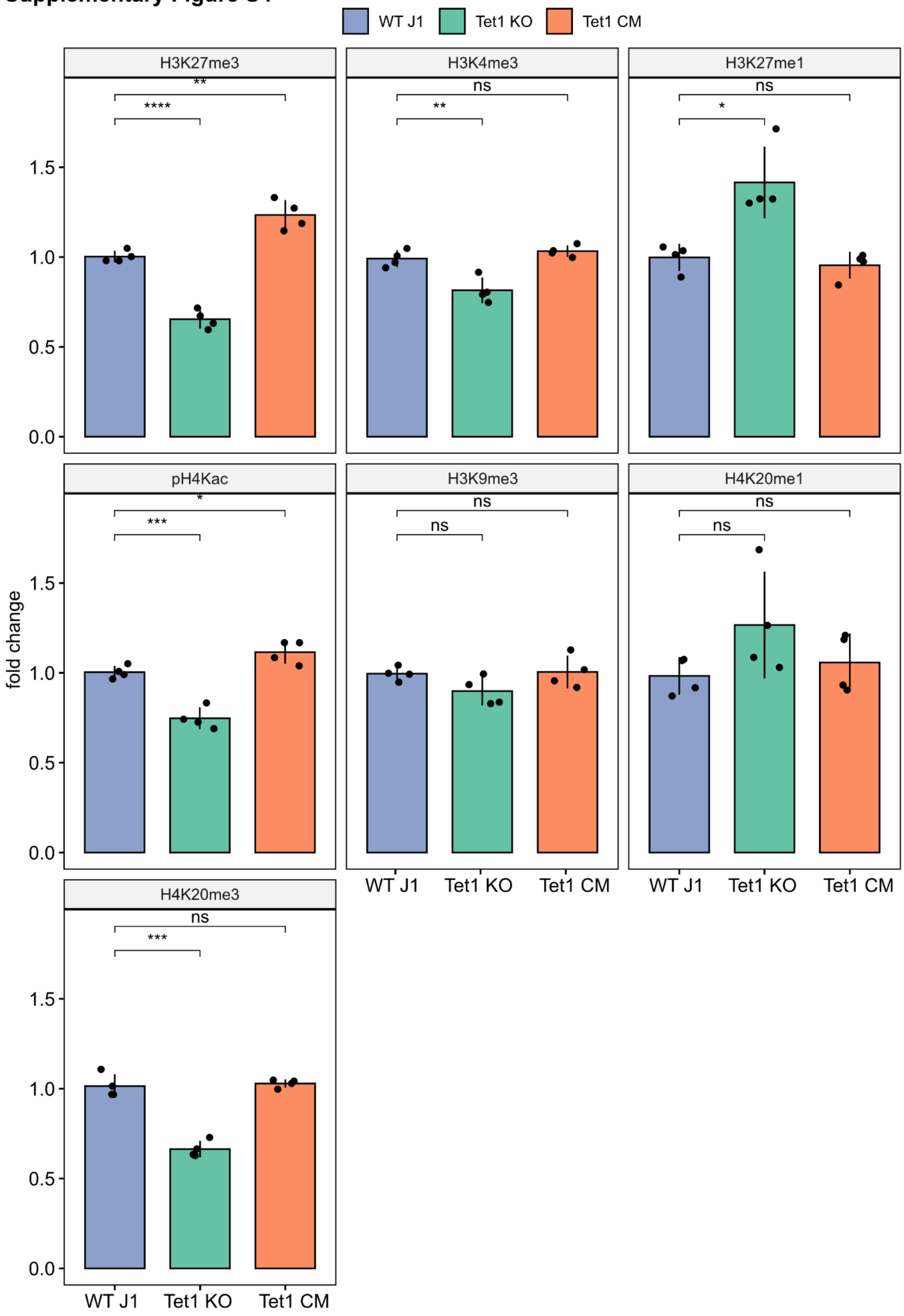

**Supplementary Figure 4. Quantification of global histone modification levels by MINUTE-ChIP.** Global quantitative MINUTE-ChIP signals for H3K27me3, H3K4me3, H3K27me1, pH4Kac, H3K9me3, H4K20me1 and H4K20me3 in WT, Tet1 KO, and Tet1 CM mESCs. Modification levels are depicted as the fold change relative to the WT. Two sided t-test: n.s. > 0.05 , *P < 0.05, **P < 0.01, ***P < 0.001, and ****P < 0.0001 (n = 4 independent replicates).

## Supplementary Figure S5

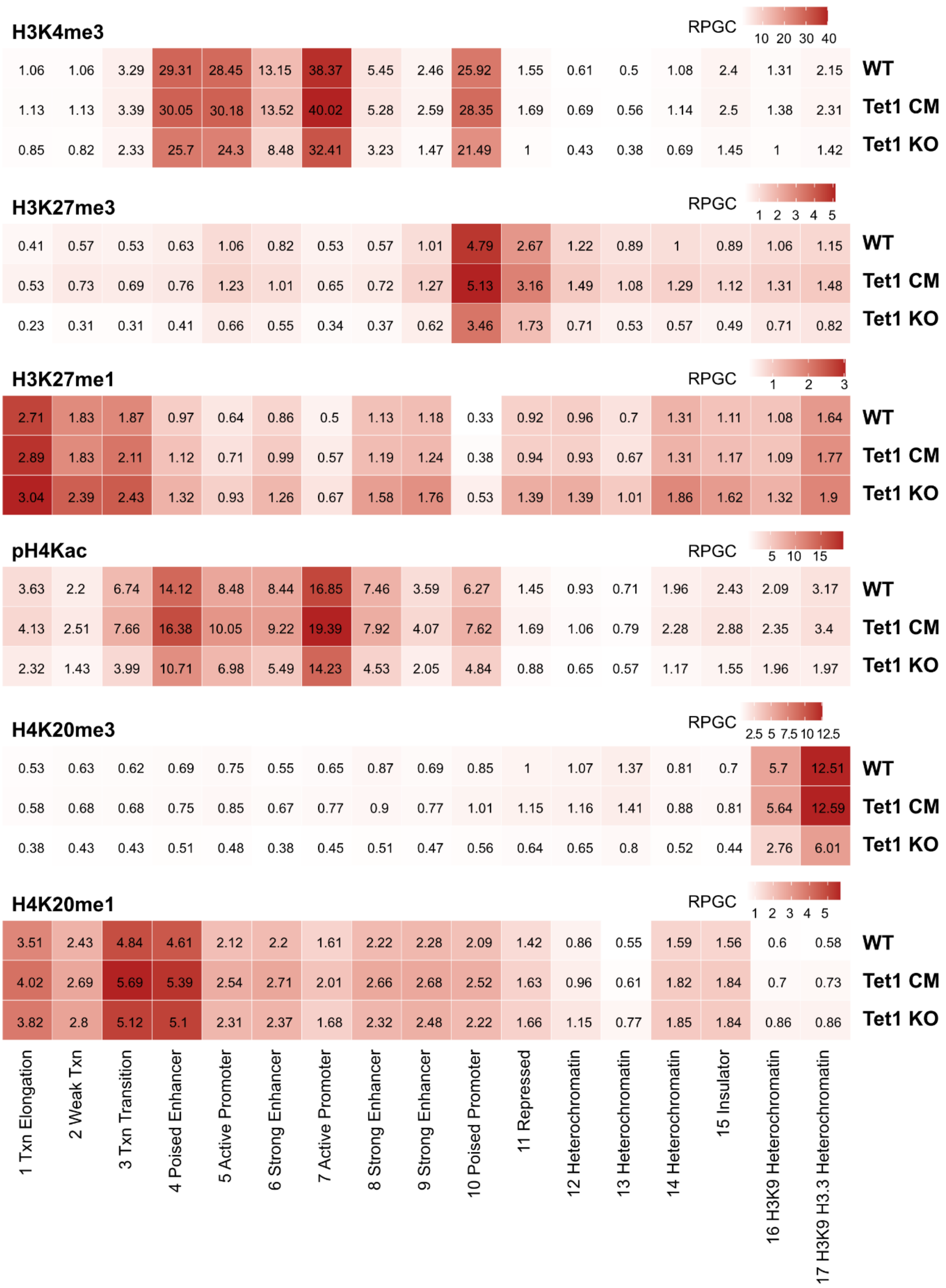


**Supplementary Figure 5. Loss of histone modifications at different chromatin regions.** ChromHMM heatmap of the quantitative ChIP-seq data comparing Tet1 KO, Tet1 CM, and WT mESCs. Depicted are the reads per genome coverage

(RPGC) of the histone modifications H3K4me3, H3K27me3, H3K27me1, pH4Kac, H4K20me1, and H4K20me3 at different chromatin regions (n = 4 independent replicates).

## Supplementary Figure 6

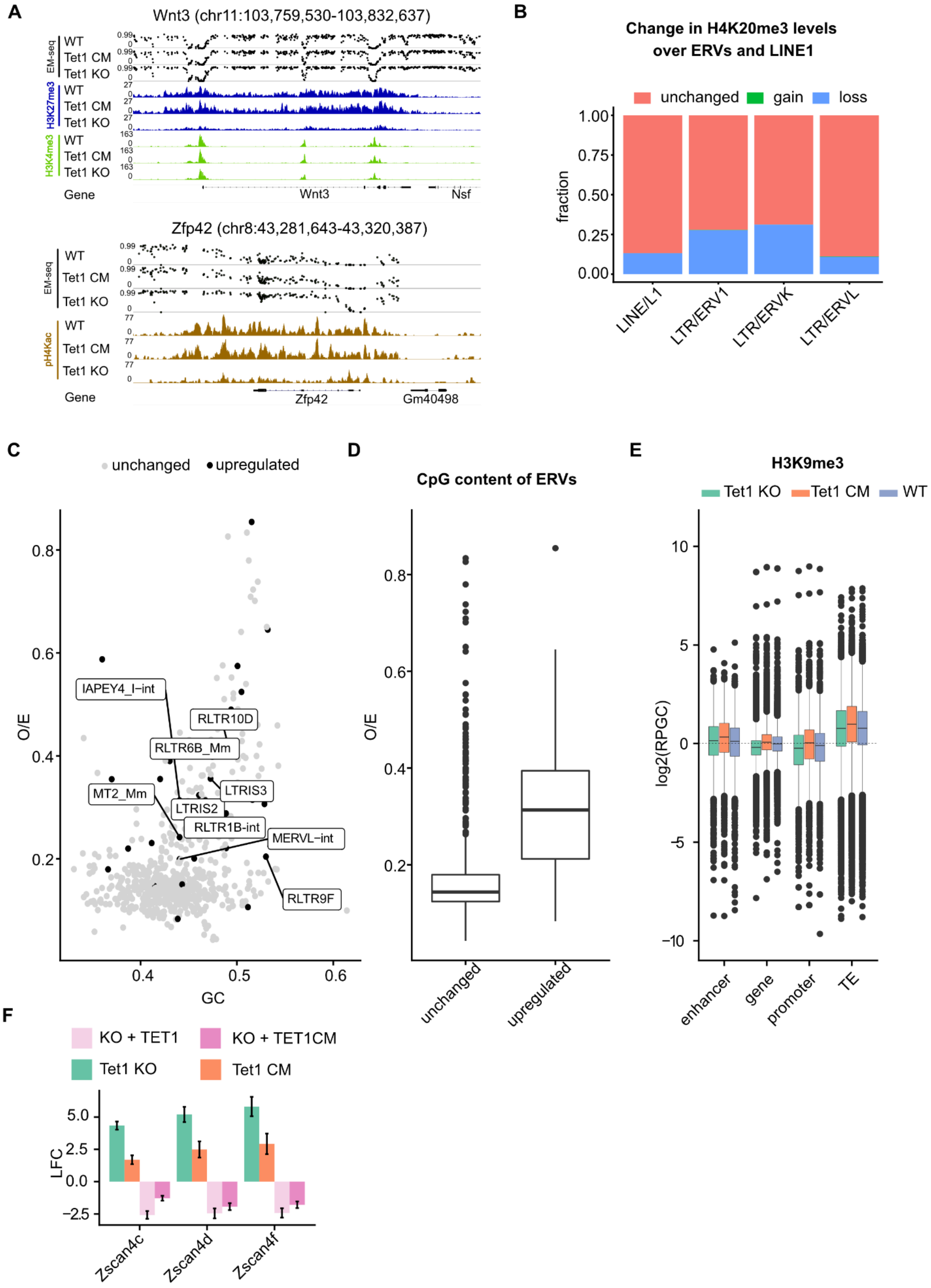

**Supplementary Figure 6. H3K9me3 and H4K20me3 loss at ERVL in Tet1 KO mESCs.** (A) Top panel: IGV genome browser view of DNA methylation (black) estimated by EM-eq, H3K27me3 (blue) and H3K4me3 (green) quantitative MINUTE-ChIP tracks at the genomic locus of the gastrulation marker *Wnt3*. Lower panel: IGV genome browser view of DNA methylation (black) estimated by EM-eq and pH4Kac (brown) quantitative MINUTE-ChIP tracks at the genomic locus of the germ cell marker *Zfp42*. Scaling of histone modifications is equal between WT, Tet1 KO and Tet1 CM mESCs. (B) Quantitative MINUTE-ChIP fraction of H4K20me3 at LINE/L1, LTR/ERV1, LTR/ERVK and LTR/ERVL elements in Tet1 KO relative to WT mESC. Displayed as the global proportions in a stacked bar plot of significant gain or loss or unchanged H4K20me3 level. LFC = log2 fold changes (n = 4 independent replicates). (C) Volcano plot depicting the observed over expected (O/E) GC content at transposable elements (TE) and color coded expression changes. Grey dots: unchanged expression of TE in Tet1 KO relative to WT mESCs. Black dots: upregulated TE in Tet1 KO relative to WT mESCs. (D) Boxplots comparing the O/E CpG content at ERVs with unchanged or upregulated expression in Tet1 KO mESCs. (E) Boxplots of H3K9me3 assessed by quantitative MINUTE-ChIP comparing Tet1 KO, Tet1 CM, and WT. Y-axis displays log2 transformed RPGC (reads per genome coverage) at enhancers, gene, promoter, and transposable element (TE). Horizontal black lines within boxes represent median values, boxes indicate the lower and upper quartiles, and whiskers indicate the 1.5 interquartile range. (F) LFC of *Zscan4c*, *d*, and *f* in Tet1 KO and Tet1 CM relative to WT mESCs and in Tet1 KO mESCs re-expressing TET1 and the TET1 SIN3A mutant relative to Tet1 KO mESCs. LFC = log2 fold changes.

## Supplementary Figure 7

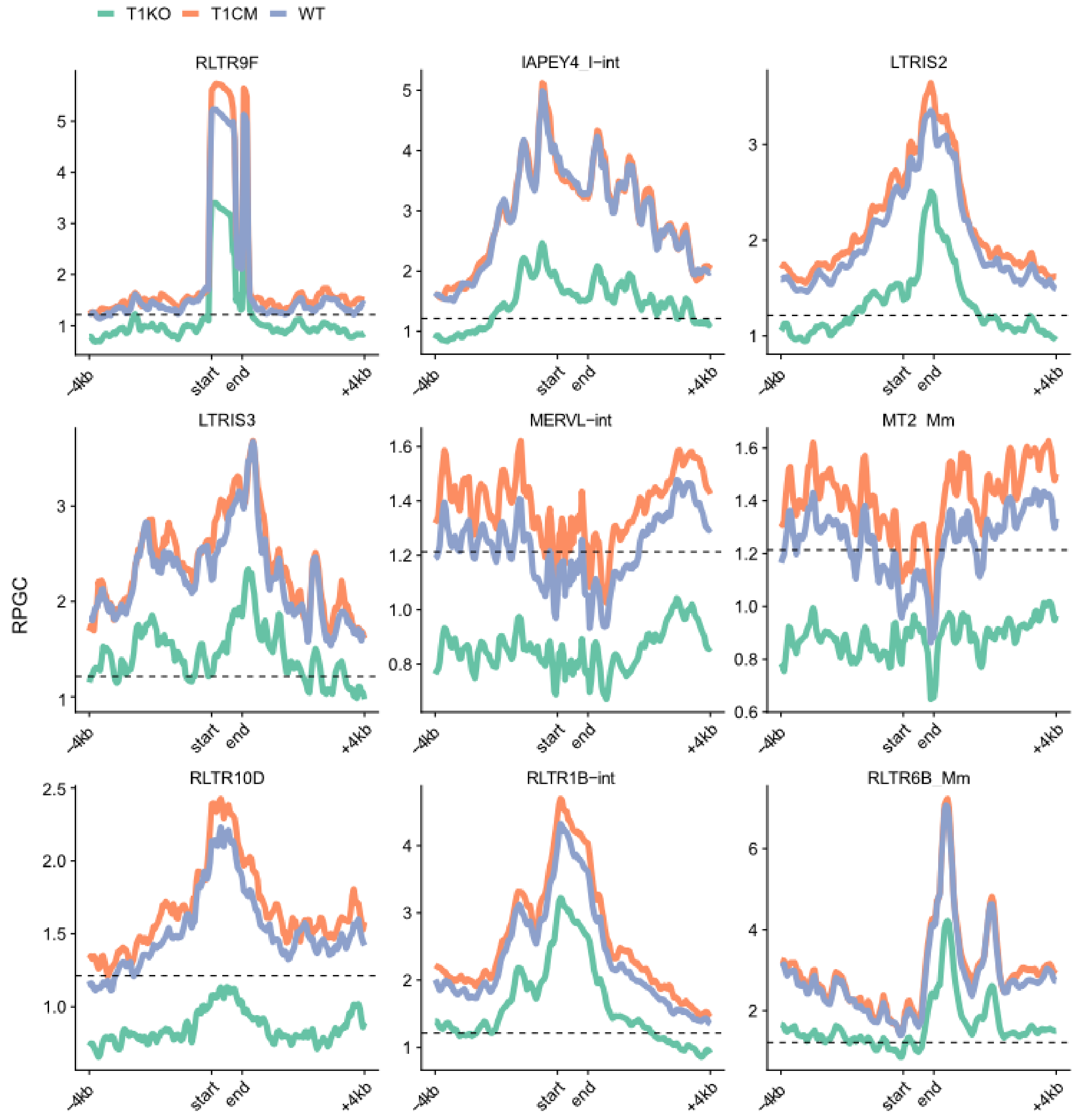


**Supplementary Figure 7. H4K20me3 abundance at ERVs.** Average quantitative MINUTE-ChIP signal of H4K20me3 at significantly upregulated ERVs in TET1 KO mESCs comparing Tet1 KO, Tet1 CM, and WT mESCs.

**Supplementary Figure 8**

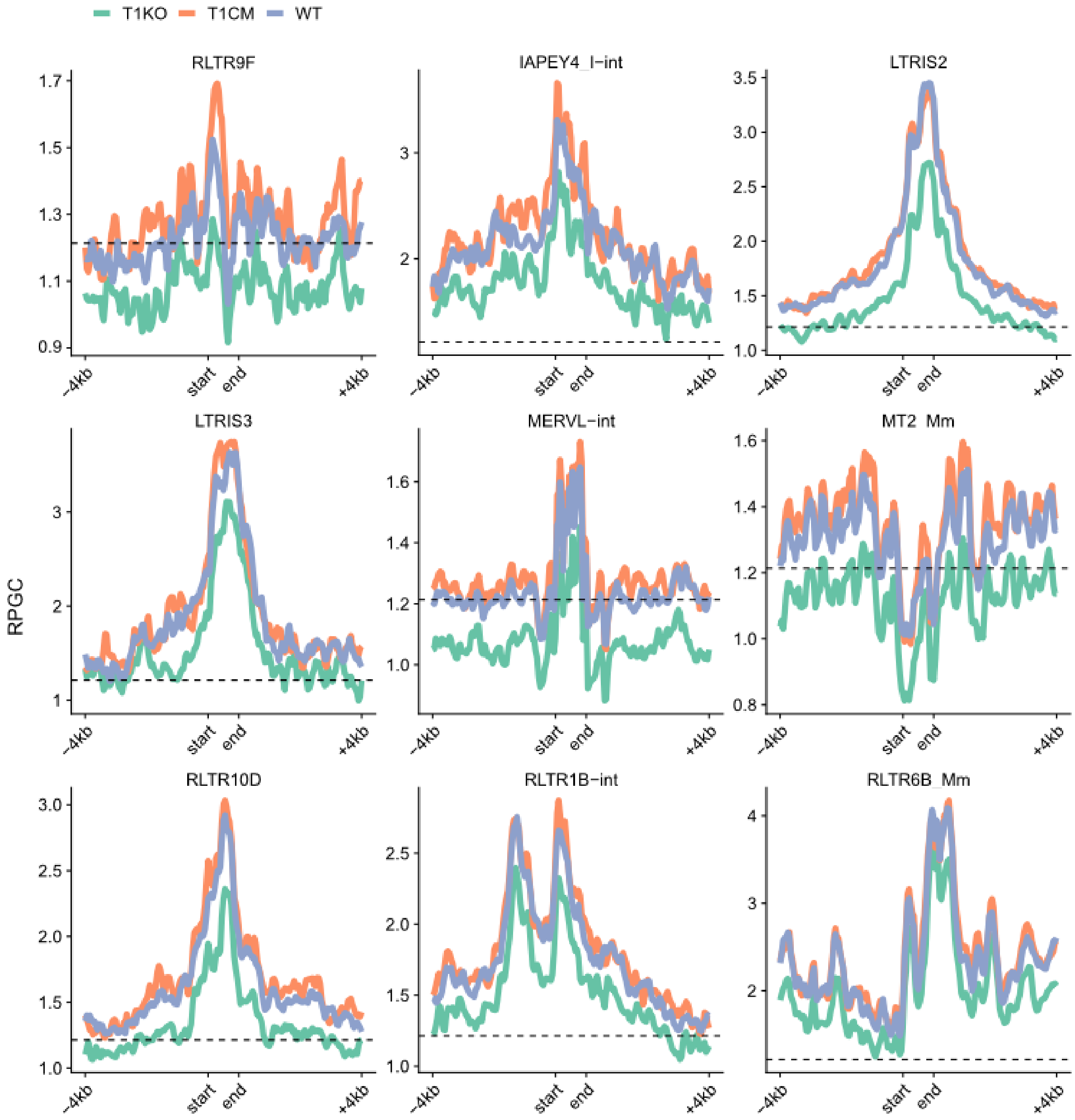


**Supplementary Figure 8. H3K9me3 abundance at ERVs.** Average quantitative MINUTE-ChIP signal of H3K9me3 at significantly upregulated ERVs in TET1 KO mESCs comparing Tet1 KO, Tet1 CM, and WT mESCs.

## Supplementary Figure 9

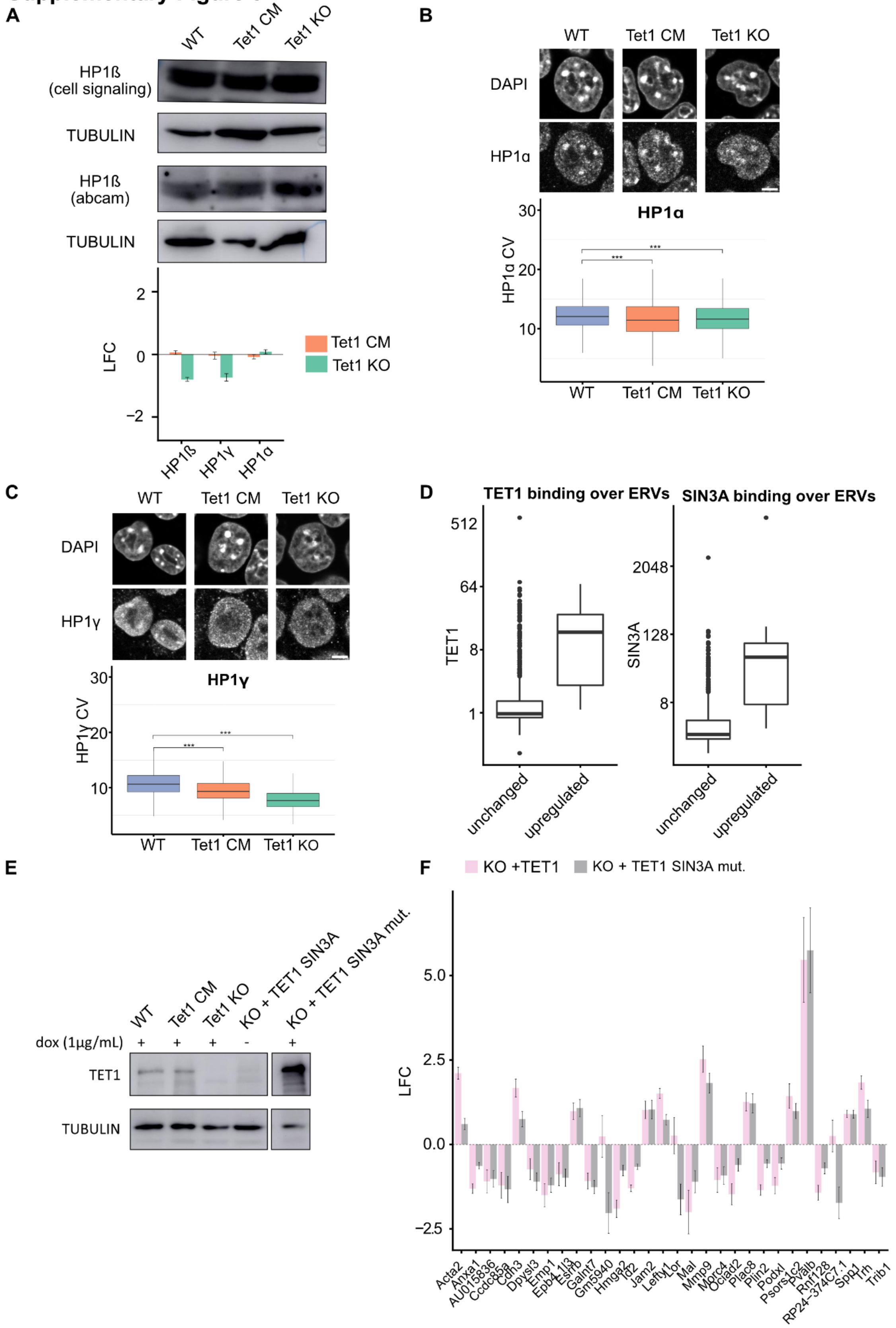

**Supplementary Figure 9. TET1 and SIN3A regulate HP1 localization and ERV repression** (A) Western blot stained for HP1β and TUBULIN as loading control in WT, Tet1 KO, and Tet1 CM mESCs using two different HP1β antibodies (Cell Signaling #8676, abcam #10478). In addition, expression levels of HP1α, β, and γ in Tet1 KO and Tet1 CM assessed by RNA-seq. Log2 fold change (LFC) relative to WT mESCs. (B) Immunofluorescence images of WT, Tet1 CM, and Tet1 KO mESC stained for DAPI and HP1α. Scale bar = 5 μm. Boxplots of the coefficient of variation (CV) calculated from HP1α immunofluorescence (IF) signal intensities, comparing WT (n = 54036), Tet1 CM (n =57990), and Tet1 KO (n = 49829). (C) Immunofluorescence images of WT, Tet1 CM, and Tet1 KO mESC stained for DAPI and HP1γ. Scale bar = 5 μm. Boxplots of the coefficient of variation (CV) calculated from HP1γ IF signal intensities, comparing WT (n = 51706), Tet1 CM (n = 58217), and Tet1 KO (n = 41610). (B+C) Images and IF intensity values were acquired and analyzed using an Operetta microscope. ANOVA + Tukey's honestly significant difference post-hoc test: ****$P$ < 0.0001. (D) Binding of TET1 and SIN3A at unchanged (n = 743) and upregulated (n = 32) ERVs in Tet1 KO mESCs using published ChIP-seq data of mESCs cultured under the same conditions (34, 55). Welch two sample t-test significant difference: *P < 0.05 (TET1 P = 0,01314, SIN3A P = 0,01436). Y-axis displays the RPGC (reads per genome coverage) of the respective protein. Horizontal black lines within boxes represent median values, boxes indicate the lower and upper quartiles, and whiskers indicate the 1.5 interquartile range. (E) Western blot stained for TET1 and TUBULIN as loading control. Comparing TET1 level in WT, Tet1 CM, Tet1 KO, and the doxycycline inducible TET1 rescue system carrying a mutation in the SIN3A interaction domain with (+) and without (-) doxycycline. (F) Expression of genes rescued by re-expressing TET1 and TET1 SIN3A in Tet1 KO mESCs. LFC relative to Tet1 KO mESCs.

**Supplementary Figure 10**

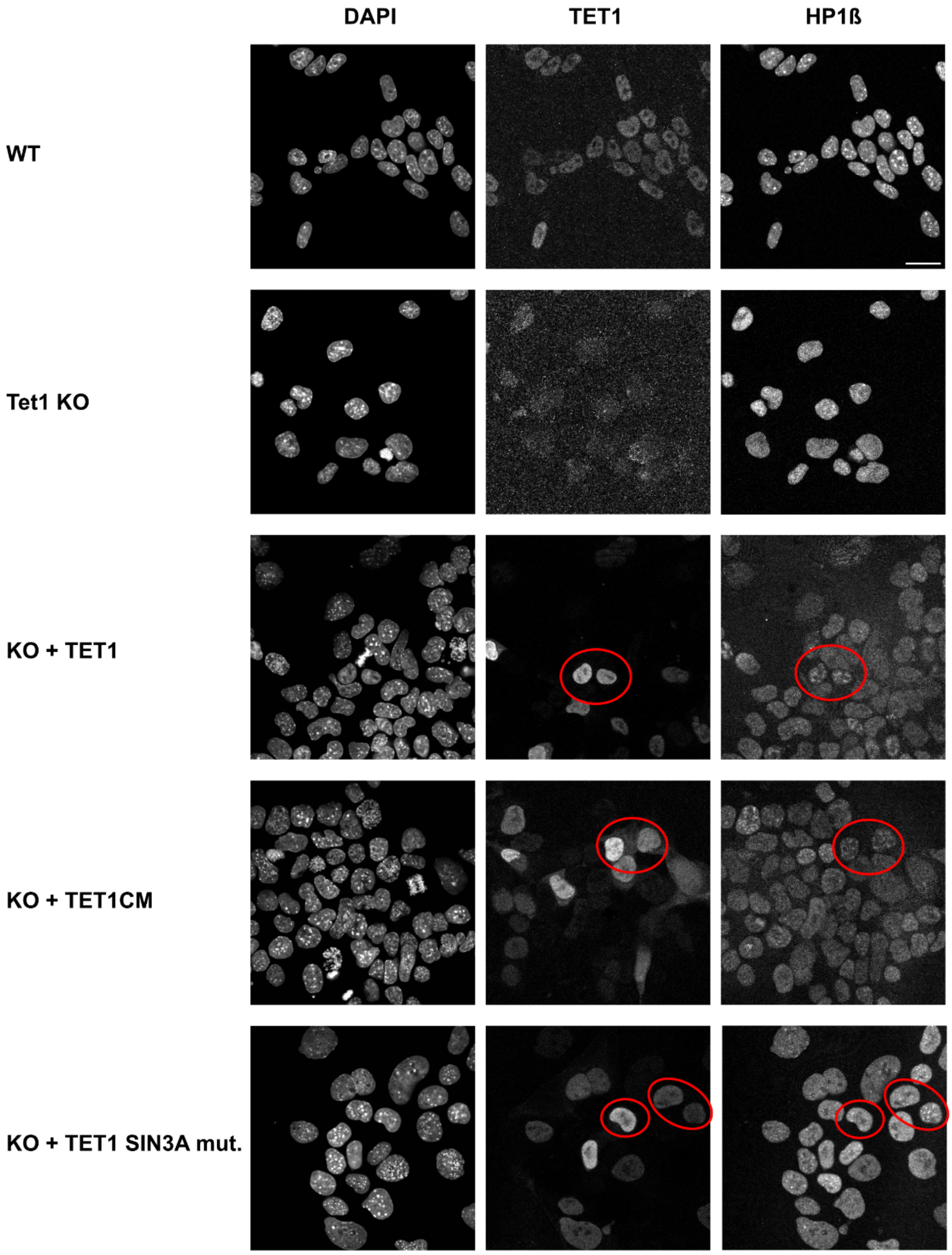


**Supplementary Figure 10. Immunofluorescence images of TET1 and HP1β stainings in TET1 rescue cell lines.** Representative immunofluorescence images of TET1 and HP1β stainings in WT, Tet1 KO, KO + TET1, KO + TET1CM and KO + TET1 SIN3A mut. corresponding to Figure 4E. Images acquired at a confocal microscope. Scale bar = 10 μM.
