# Supplementary material for: TET1 regulates gene expression and repression of endogenous retroviruses independent of DNA demethylation
Source: Nucleic Acids Res. 2022 Jul 29;50(15):8491–511. doi: 10.1093/nar/gkac642 (PMC9410877; doi:10.1093/nar/gkac642)
Supplement: gkac642_Supplemental_Files [file gkac642_supplemental_files.zip › Supplementary Tables Legends.docx]

**Supplementary Table 1.** List of Gene Ontology (GO) enrichment analysis with ID, Description, setSize, enrichmentScore, NES, pvalue, p.adjust, qvalues, rank, leading_edge, and core_enrichment of RNA-seq data comparing Tet1 KO and WT mESCs.

**Supplementary Table 2.** EM-seq library information of WT, Tet1 KO, and Tet1 CM mESCs with sample_id, genotype, total reads, mapped reads, CpGs covered > 5x, % mapped reads, and conversion efficiency.

**Supplementary Table 3.** List of p.value and p.adj calculated for quantitative MINUTE-ChIP signals of H3K27me3, H3K4me3, H4K20me3, and pH4Kac comparing Tet1 KO, Tet1 CM, and WT at enhancer, gene body inactive/active, promoter inactive/active, and transposable element (TE).

|  |  |  |  |  |  |  | |
| --- | --- | --- | --- | --- | --- | --- | --- |
